# Multimodal control of Cas13d activity through domain insertion at an allosteric hotspot

**DOI:** 10.1038/s41467-026-73645-5

**Published:** 2026-06-03

**Authors:** Liyuan Zhu, Long T. Nguyen, Alexandra G. Bell, Tom Krebel, Kara M. Gillmann, Qinhao Cao, Harrison Oatman, Jack Hariri, Andreas Möglich, Cameron Myhrvold, Jared E. Toettcher

**Affiliations:** 1 Department of Chemistry, Princeton University, Princeton, NJ USA; 2 Omenn-Darling Bioengineering Institute, Princeton University, Princeton, NJ USA; 3 Department of Molecular Biology, Princeton University, Princeton, NJ USA; 4 Photobiochemistry Group, Department of Chemistry, University of Bayreuth, Bayreuth, Germany; 5 Lewis Sigler Institute for Integrative Genomics, Princeton University, Princeton, NJ USA; 6 Department of Chemical and Biological Engineering, Princeton University, Princeton, NJ USA

**Keywords:** Synthetic biology, Chemical biology, CRISPR-Cas systems

## Abstract

CRISPR-Cas13d RNA nucleases are powerful tools for programmable RNA targeting. A light-controlled RNA nuclease could be transformative by enabling researchers to selectively knock down transcripts at desired positions in a cell or tissue or at timepoints of interest. Here, we develop a set of RfxCas13d tools that can be multimodally controlled by either light or small molecule addition. By screening an RfxCas13d library containing insertions of the AsLOV2 photoswitchable domain, we identify an OptoCas13d-off variant that induced target RNA cleavage in the dark and switched to an inactive state under blue light. We show that the same allosteric hotspot can be exploited to generate an OptoCas13d-on with an inverted light response and a ChemoCas13d that is activated by rapamycin analogs, enabling knockdown of endogenous mRNA and protein targets. Overall, our study shows that engineered allostery can produce stimulus-controlled Cas13d variants to modulate RNA with high spatial and temporal precision.

## Abstract (web summary)

The Cas13 family of RNA-guided RNA nucleases are powerful tools for targeted RNA perturbations, but their activity is difficult to control in space and time. Here, authors present a set of engineered optogenetic and chemogenetic Cas13 variants to precisely control RNA cleavage.

## Introduction

Cas13, an RNA-targeting CRISPR effector protein, provides a versatile platform for programmable RNA cleavage, detection, editing and imaging^1–5^. *Ruminococcus flavefaciens* Cas13d (RfxCas13d) has been identified as one of the most efficient Cas13d orthologs for transcript knockdown in diverse cellular and animal contexts^1–4^. RfxCas13d binds to a cognate CRISPR RNA (crRNA). Pairing of the RfxCas13d-crRNA complex and a target RNA activates Cas13d nuclease activity, resulting in cleavage of the target RNA. Variations of this approach include using a catalytically dead Cas13d mutant fused to an RNA editing enzyme (e.g., ADAR2) to drive target RNA modification instead of degradation^5^.

One limitation with many current Cas13d tools is that they are constitutively active, performing RNA degradation or modification whenever Cas13, the crRNA, and the target RNA are present. More precise control over Cas13d activity would offer several advantages. First, RNA expression is precisely controlled in space and time to regulate functions both within cells (e.g., RNA trafficking in neurons) and across a tissue (e.g., gene expression in a developing embryo)^6–9^. Therefore, the development of a spatiotemporally controllable Cas13d system would enable more precise investigations into RNA function and regulation with high resolution. Additionally, recent studies have highlighted that Cas13d exhibits *trans*-cleavage activity: cleavage of RNAs other than the transcript of interest^10,11^. More precise control over the concentration of active Cas13d species could mitigate the collateral effects of *trans* cleavage and reduce cytotoxicity^12^.

Recent work has led to the development of split Cas13d systems, including the padCas13^13^, CRISTAL (Control of RNA with Inducible SpliT Cas13d Orthologs and Exogenous Ligands)^14^, and an abscisic acid (ABA)-inducible split dCas13b system^15^. These approaches all rely on dual-component systems that function via the reconstitution of split proteins. Split proteins are powerful but can have several drawbacks, including the requirement to express two protein components that may each have reduced stability compared to the full-length protein. Split enzymes can also exhibit residual binding and leaky activity, as well as reduced catalytic activity once complemented^16,17^, which could limit the overall efficacy of target cleavage. Perhaps due to these drawbacks, the only light-controlled Cas13d tool developed to date performs well as a nuclease-dead fusion to the ADAR base editor, whereas a catalytically active variant only achieves weakly light-switchable RNA knockdown^13^.

Here, we developed several single-domain, stimulus-controllable Cas13d nucleases based on domain insertion and allosteric control. Screening a library of AsLOV2 insertions into RfxCas13d revealed a single insertion site that conferred photoswitchable degradation of reporter RNA. Through subsequent optimization and insertion of distinct regulatory domains, we generated a light-inhibited variant via AsLOV2 insertion^18,19^ (OptoCas13d-off), a light-activated variant through LightR insertion^20^ (OptoCas13d-on), and a rapamycin-inducible variant incorporating the UniRapR domain^21^ (ChemoCas13d). We further validated the efficacy of ChemoCas13d and OptoCas13d-on in mediating stimulus-inducible knockdown of endogenous transcripts, highlighting its potential as a powerful tool for studying gene function with high spatiotemporal resolution. These findings underscore the effectiveness of combinatorial screening approaches in identifying allosteric hotspots within structurally complex proteins, paving the way for future advances in optogenetic, chemically inducible, and other modalities of allosterically controlled protein engineering.

## Results

### Construction of RfxCas13d-AsLOV2 random insertion library and reporter landing pad cell line

In recent years, insertion of an optogenetic or chemogenetic domain into a protein of interest has gained prominence as a technique for creating stimulus-switchable proteins^19,20,22–24^. However, finding suitable insertion sites can be difficult, particularly for large proteins where structural information is incomplete. We recently reported that domain insertion profiling^25^ and screening could be used for unbiased discovery of suitable insertion sites^26^. We generated a library in which the AsLOV2 photoswitchable domain was inserted at many random positions into a Gal4-VP64 transcription factor and screened variants for photoswitchable gene expression, yielding an OptoGal4 transcription factor with a > 150-fold change in gene expression between dark and illuminated states^26^. However, domain insertion has not yet been reported for Cas13d RNA-editing enzymes, a challenging family whose members are large (often >1000 amino acids) and possess multiple functional domains.

**Fig. 1:**
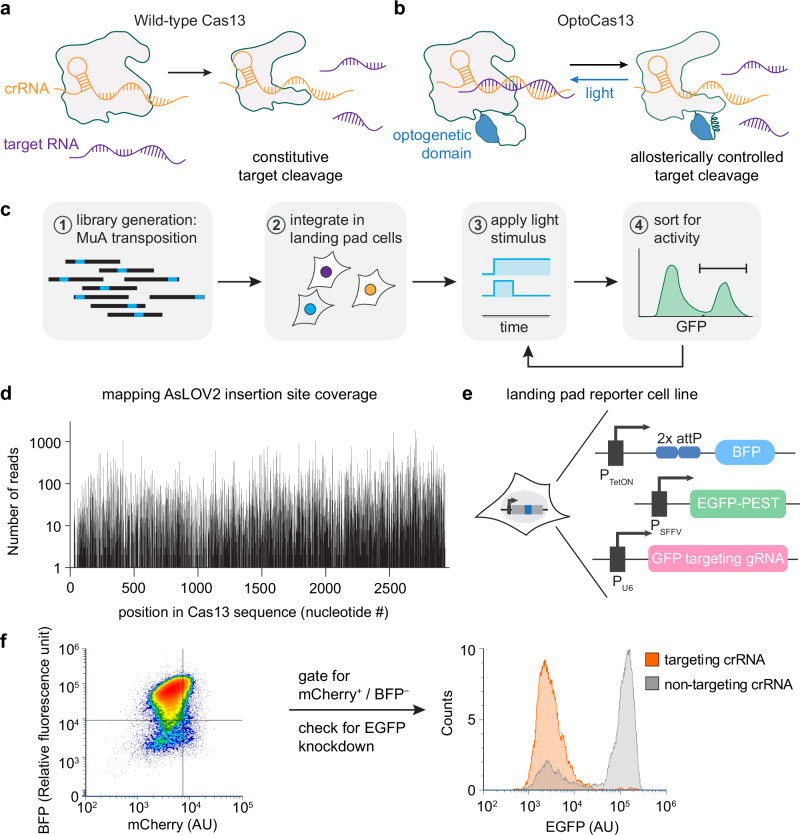
Engineering approach for generating optogenetically controllable RfxCas13d variants. **a, b** Comparison of the mechanism of a wild-type RfxCas13d cleavage (in (**a**)) and an allosterically controlled RfxCas13d via light-sensitive domain insertion (in (**b**)). **c** Schematic of the workflow for generating RfxCas13d-AsLOV2 insertion library and incorporating them into landing pad reporter cell lines for selections. **d** Coverage of random AsLOV2 insertion sites in between amino acid residues within RfxCas13d through next-generation sequencing analysis. **e** Composition of a landing pad reporter cell line. This cell line contains a single recombination site with the BFP gene in frame for negative selection. This recombination site acts as a landing pad for the incorporation of the RfxCas13d-AsLOV2 insertion library. The landing pad cell line also contains a reporter EGFP gene and a crRNA targeting EGFP mRNA. **f** Successful incorporation of the RfxCas13d-AsLOV2 insertion library results in displacement of the BFP gene by mCherry. BFP- negative and mCherry-positive cell population was sorted via fluorescence-activated cell sorting (FACS). EGFP knockdown was confirmed in this sorted subpopulation. Source data are provided as a Source Data file.

We first set out to construct a comprehensive library of AsLOV2 insertions into the RfxCas13d protein scaffold, and to establish a cell line in which to test the efficacy of this library for RNA knockdown in mammalian cells (Fig. 1a, b). For library construction, we employed a well-adopted approach for generating a random domain insertion library, referred to as DIP-Seq^25^ (Fig. 1c). This approach utilizes a Mu transposase to randomly insert the chloramphenicol resistance (CmR) gene into a target gene of interest. The chloramphenicol-resistant library is then inserted into an expression vector, followed by replacement of CmR with the domain of interest. We modified the original DIP-Seq protocol to adjust for the insertion of a small domain into large target proteins and to account for the presence of BsaI sites in the destination vector (Supplementary Fig. 1). After moving the library of AsLOV2-inserted RfxCas13d variants into the final landing pad recombination vector, we performed next-generation DNA sequencing of the library and showed that our library covers at least 91% of possible insertion sites (Fig. 1d). The resulting library was then integrated into a reporter cell line for follow-up screening.

We next created a HEK293T landing pad reporter cell line where the activity of RfxCas13d could be assessed by EGFP knockdown, allowing for the measurement and screening of RfxCas13d library variants through fluorescence-activated cell sorting (FACS)^27,28^ (Fig. 1e). The reporter cell line was constructed to include both a constitutively expressed, destabilized EGFP reporter (EGFP-PEST) and an EGFP mRNA-targeting CRISPR RNA (crGFP), so that the introduction of a functional RfxCas13d variant could cleave EGFP mRNA and reduce the cell’s overall EGFP expression. This landing pad cell line allows for the generation of a pooled library of RfxCas13d insertion variants, with each cell expressing a single variant from a defined genomic locus in the cell (Fig. 1e). By sorting cells that are mCherry-positive and BFP-negative after transfection, we could enrich only those cells with successful landing pad incorporation. A ~100-fold decrease in EGFP expression was observed in RfxCas13d-expressing reporter cells compared to an analogous cell line expressing a non-targeting crRNA, consistent with robust RfxCas13d/crRNA-targeted EGFP degradation in this cell line (Fig. 1f).

### Domain insertion at the QK634 allosteric hotspot confers light-switchable Cas13d activity

**Fig. 2:**
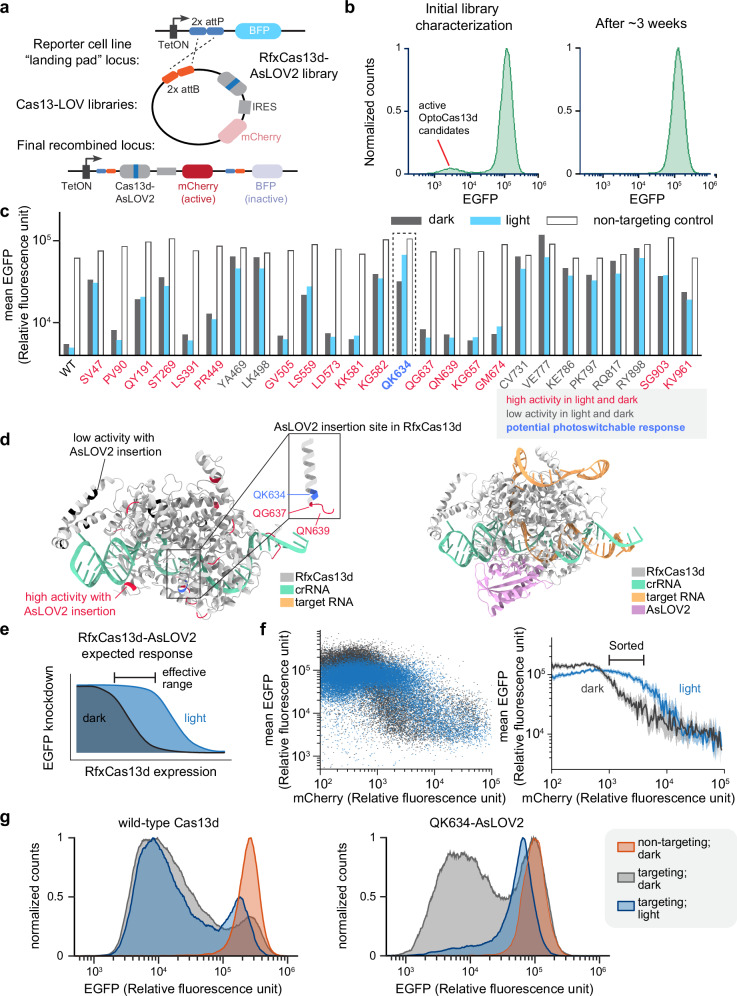
Screening RfxCas13d-AsLOV2 variants for light switchable behavior. **a** Schematic of successful recombination of the RfxCas13d-AsLOV2 insertion library into the landing pad reporter cell line. **b** Histogram showing the loss of EGFP knockdown during routine cell culture, indicating a potential selection against active variants. **c** Individual candidates were tested for photoswitchability. Many variants either exhibited loss of function due to domain insertion (red text) or no switchable behavior (black text). Site QK634 (blue text) was identified as a potential candidate. **d** Left: predicted structure of wild-type RfxCas13d / crRNA complex with insertion sites from (**b**) colored according to photoswitchable activity. Right: predicted structure of RfxCas13d-AsLOV2^QK634^ / crRNA / target RNA complex. Structure prediction was performed using AlphaFold3 (see “Methods”) and visualized with ChimeraX^41–43^. **e** A schematic of predicted RfxCas13d-AsLOV2^QK634^ activity as a function of expression level and illumination conditions. **f** Flow cytometry of EGFP fluorescence (indicating RfxCas13d functionality) and mCherry fluorescence (indicating RfxCas13d expression) under light and dark conditions. Left panel: single-cell responses. Right panel: error bars show the mean ± 95% confidence interval for single cells binned from (**f**). Data shown is from one representative of *n* = 3 biological replicates. **g** Flow cytometry histograms of EGFP fluorescence after transfecting cells expressing RfxCas13d-AsLOV2^QK634^ with EGFP-targeting or non-targeting crRNA in the indicated illumination conditions. Data shown is from one representative of *n* = 3 biological replicates. Source data are provided as a Source Data file.

We introduced the RfxCas13d-AsLOV2 library into the landing pad cell line and set out to screen the library for EGFP knockdown in both light and dark conditions (Fig. 2a). We observed that after the library was initially expressed, many variants exhibited high EGFP levels, with a small subpopulation still successfully inducing EGFP knockdown. However, a major challenge arose—these knockdown-competent populations were lost after approximately three weeks in culture, indicating a potential selection against continuous Cas13d activity in the landing pad cell line (Fig. 2b). We hypothesized that constitutive expression of RfxCas13d may have been toxic in this cell line due to the high expression level of RfxCas13d and/or EGFP target RNA driving *trans* cleavage activity^12^.

Faced with this setback, we hypothesized that the set of highly active variants that were lost during culture might also include variants with photoswitchable activity, since these variants tolerated AsLOV2 insertion while remaining active in the dark. To identify the insertion sites represented by these variants, we performed next-generation sequencing of the library before and after cell incorporation. While most insertion sites were present at similar proportions in both libraries, we identified 27 sites that were strongly depleted after mammalian cell integration and culture (Supplementary Fig. 2). We selected these variants for individual testing to assess their knockdown activity under dark or light conditions. Among the 26 OptoCas13d variants tested (one variant was not recovered during cloning), 16 retained constitutive high activity under both dark and light conditions, and 9 showed low activity in both dark and light. Notably, we identified one OptoCas13d variant with an insertion at site QK634 (AsLOV2 inserted between Glu634 and Lys635), which exhibited stronger EGFP knockdown in the dark compared to the light (Fig. 2c, Supplementary Fig. 3).

To better understand the structural context of each insertion site, we modeled both binary (RfxCas13d:crRNA) and tertiary (RfxCas13d:crRNA:target RNA) complexes using AlphaFold3^29^ (Fig. 2d, Supplementary Fig. 4). The AlphaFold3 predictions indicated that some insertions were positioned within well-folded structural motifs (e.g., within α-helices or β-strands of the protein), providing a likely explanation for why AsLOV2 insertion in these positions could break Cas13d functionality. We then mapped the potential allosteric hotspot, QK634 (highlighted in green), onto the predicted structure of RfxCas13d. This site is situated within the Helical-2 linker domain, which bridges two catalytic domains. Structural predictions indicate that QK634 is surface-exposed and positioned spatially distant from the active sites. A closer examination of the QK634 position revealed that it resides at the transition point between an unstructured loop, where AsLOV2 insertion does not disrupt Cas13d functionality in either dark or light conditions (see e.g., the QG637 and QN639 sites), and a well-folded α-helix, where AsLOV2 insertion completely abolishes enzymatic activity. This unique positioning may enable QK634 to accommodate AsLOV2 insertion while effectively transmitting the conformational changes induced by light to regulate overall OptoCas13d functionality (Fig. 2d, Supplementary Fig. 4).

We next sought a more quantitative picture of light-switchable EGFP knockdown in the QK634 RfxCas13d variant, henceforth termed OptoCas13d-off. We reasoned that the conformational switch might exhibit concentration dependence, switching the threshold of Cas13d expression at which potent EGFP knockdown would be observed between light and dark cases (Fig. 2e). We analyzed EGFP reporter expression in cells transfected with an OptoCas13d-off IRES mCherry construct, so that the OptoCas13d-off expression level could be indirectly visualized using mCherry expression. Indeed, we observed that EGFP knockdown in light and dark depended on the Cas13d expression level. This experiment also led us to identify a specific mCherry expression range where EGFP knockdown occurred in the dark but not in cells illuminated with blue light (Fig. 2f).

We next generated a clonal EGFP-expressing cell line harboring either wild-type Cas13d or the OptoCas13d-off system and sorted each for the specific mCherry expression level, where we observed light-inducible knockdown (Supplementary Fig. 5). Unlike the landing pad reporter cell line (Fig. 2a), which stably integrates both EGFP and an EGFP-targeting crRNA, this EGFP-expressing cell line does not express the crRNA, ensuring that Cas13d incorporation would not lead to constitutive mRNA cleavage and affect cell viability. We observed comparable EGFP knockdown in dark conditions in both wild-type and OptoCas13d-off cells, whereas knockdown was nearly absent in both lines upon transfection with a non-targeting crRNA (Fig. 2g). Crucially, light blocked EGFP knockdown in OptoCas13d-off cells but not wild-type Cas13d cells (Fig. 2g). We observed some heterogeneity in EGFP knockdown from both wild-type and OptoCas13d-off cells, suggesting that there was variability in knockdown efficiency between cells that might arise from variability in crRNA transfection or Cas13d expression. Collectively, these findings demonstrate that inserting AsLOV2 at the QK634 site in RfxCas13d yields a light-inhibited Cas13d variant.

### Development of multimodal RfxCas13d variants with alternative regulatory domains

Although our OptoCas13d-off system confers potent light-dependent regulation of RNA cleavage, we can envision applications where alternative stimulus modalities would be preferable to induce Cas13d activity. Fortunately, the principle of allosteric control is quite modular, permitting multiple light- and drug-switchable domains to be in principle inserted at the same position. To enable multimodal control of RfxCas13d activity, we replaced the AsLOV2 domain at the QK634 site with alternative domains capable of undergoing conformational changes in response to specific stimuli.

**Fig. 3:**
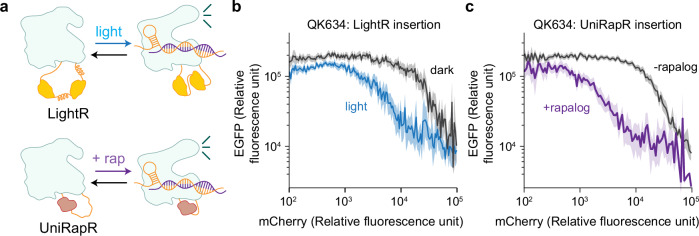
Expansion of controllable RfxCas13d variants with alternative regulatory domains. **a** Schematic depicting LightR and UniRapR domains inserted between residues QK634 in RfxCas13d. **b**, **c** Flow cytometry data of EGFP fluorescence from single cells expressing either RfxCas13-LightR^QK634^ (left) or RfxCas13d-UniRapR^QK634^ (right). Error bars (shaded areas) show the mean ± 95% confidence interval for single cells. Data shown is from one experiment representative of *n* = 3 biological replicates. Source data are provided as a Source Data file.

To demonstrate the modularity of our approach, we selected two domains—UniRapR and LightR—both of which have been previously reported to confer stimulus-inducible properties to target proteins upon insertion^20,21^. UniRapR is a single-chain protein composed of a modified insertable FKBP subdomain fused to two helices derived from FRB. Upon rapamycin binding, UniRapR undergoes an intramolecular conformational switch by bringing its two subdomains into proximity. In contrast, LightR consists of two VVD domains linked by a flexible peptide linker^20^. VVD is a light-sensitive domain that exists as a monomer in the absence of light but undergoes homodimerization upon light exposure, leading to an overall conformational change in LightR. Structurally, AsLOV2 exists in a closed conformation in the dark, with its two terminal loops positioned proximally. Light exposure induces undocking of these loops, transitioning AsLOV2 into an open conformation^30^. In contrast, UniRapR adopts an open conformation under normal conditions but undergoes closure upon rapamycin addition, while LightR remains open in the dark and transitions to a closed conformation in response to light. Based on these properties, we hypothesized that substituting AsLOV2 with UniRapR or LightR in RfxCas13d would render the enzyme responsive to rapamycin or light, respectively, but with stimulus-dependent activation rather than inhibition as is the case for AsLOV2 (Fig. 3a).

To test this hypothesis, we transfected an EGFP-expressing stable cell line with RfxCas13d variants tagged with IRES-mCherry and co-transfected an EGFP mRNA-targeting crRNA. As anticipated, insertion of LightR into QK634 conferred light-induced activation of RfxCas13d (termed OptoCas13d-on; Fig. 3b), whereas UniRapR insertion resulted in rapamycin-dependent activation (termed ChemoCas13d; Fig. 3c). As in the case of OptoCas13d-off, both variants were only stimulus sensitive at intermediate expression levels, although this range was larger for OptoCas13d-on and ChemoCas13d compared to the original OptoCas13d-off, possibly due to a larger stimulus-induced conformational change of these variants. Overall, these experiments demonstrate that the OptoCas13d and ChemoCas13d tools are single-protein systems with potent, stimulus-switchable, and programmable RNA cleavage.

### Biochemical and cellular assays reveal modest effects on Cas13-RNA ternary complex formation

We next set out to gain insight into the mechanism of light-gated RNA cleavage using biochemical assays. We reasoned that three different processes could in principle be regulated by the photoswitchable domain: RfxCas13d:crRNA binary complex formation, RfxCas13:crRNA:target RNA ternary complex formation, or RfxCas13d cleavage of the target RNA.

**Fig. 4:**
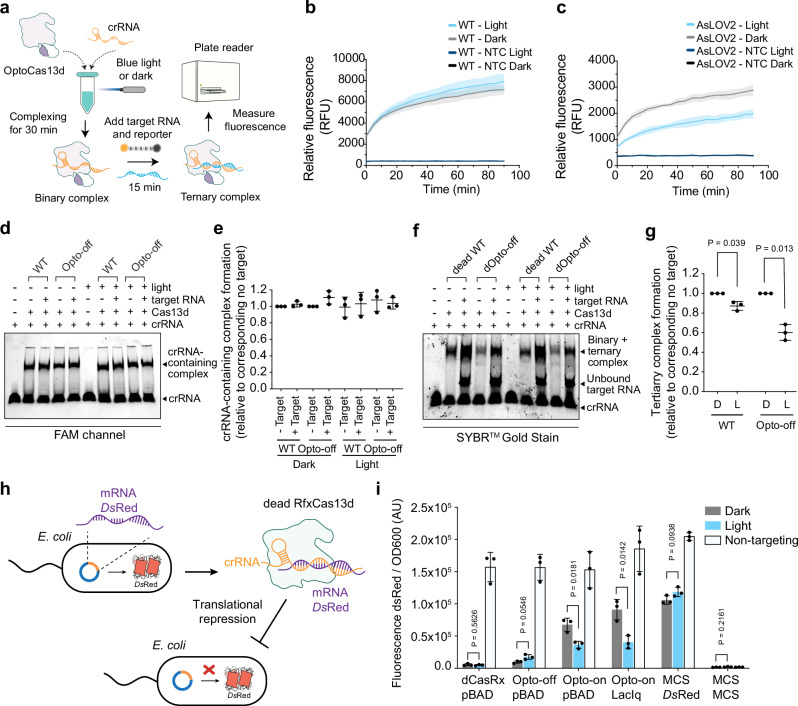
In vitro biochemical characterization of OptoCas13d-off. **a** Schematic of the trans-cleavage assay workflow with the incorporation of a light treatment step. **b**, **c** Comparison of in vitro cleavage activity of RfxCas13d (WT; in (**b**)) and OptoCas13d-off (AsLOV2; in (**c**)) in dark and light conditions. NTC: non-targeting control RNA. Shaded error bars indicate mean ± SD for *n* = 3 biological replicates. **d** EMSA assay of complexation of RfxCas13d (WT) and OptoCas13d-off (Opto-Off) with FAM-labeled crRNA in dark and light conditions. **e** Quantification of band intensity in (**d**) for *n* = 3 biological replicates. **f** EMSA assay of binary and tertiary complexation of dRfxCas13d (dead WT) and dOptoCas13d-off (dOpto-off) in dark and light conditions. **g** Quantification of band intensity in (**f**) for *n* = 3 biological replicates. **h** Schematic for dRfxCas13d-mediated translational repression in *E. coli* using *Ds*Red as a fluorescent reporter. **i** Both dOptoCas13d-off and dOptoCas13d-on exhibit light-dependent changes in translational repression. dOptoCas13d-on performs slightly better when constitutively expressed from the LacI^q^ promoter rather than the inducible pBAD. MCS denotes an empty vector control. Data are presented as mean ± S.D. from *n* = 3 biological replicates. Statistical significance was assessed using a two-tailed unpaired *t*-test. Source data are provided as a Source Data file.

We expressed and purified active and catalytically inactivated mutants of wild-type RfxCas13d and OptoCas13d-off in *E. coli*. First, we performed in vitro *trans*-cleavage assays, as previously described^30^, to determine if light-dependent cleavage could also be observed in vitro. For consistency, we used the same EGFP crRNA and synthetic mRNA fragment to assess in vitro cleavage. *Trans*-cleavage activity for wild-type RfxCas13d and OptoCas13d-off was measured immediately after exposing the RNP complex to blue (~465 nm) light or dark treatment, followed by the addition of target RNA (Fig. 4a). Notably, we noticed a substantial reduction in relative fluorescence for OptoCas13d-off under blue light compared to its dark treatment counterpart, corroborating the same observation for EGFP knockdown in HEK293T cells. On the other hand, the wild-type RfxCas13d exhibited negligible differences in fluorescence intensity between dark and blue light conditions (Fig. 4b, c). These data confirm that OptoCas13d-off retains photoswitchable cleavage activity in vitro.

To test whether light might regulate the formation of OptoCas13d-off binary or tertiary RNA complexes, we turned to electrophoretic mobility shift assays (EMSAs) to measure changes in RNA-protein binding. We incubated OptoCas13d-off with an EGFP-targeting crRNA whose 3’ end was labeled with a FAM fluorophore to measure changes in crRNA:Cas13d binary complex formation and found that blue light illumination had no effect on the fraction of crRNA:Cas13d binary complexes for either wild-type RfxCas13d or OptoCas13d-off (Fig. 4d, e, Supplementary Fig. 6). For analysis of ternary complex formation, we purified dead Cas13d variants of (dRfxCas13d and dOptoCas13d-off) harboring R239A/H244A/R858A/H863A mutations that allow for RNA binding but not cleavage. We incubated each Cas13d variant with an EGFP-targeting crRNA (for binary complex formation or the crRNA and a cognate 107-base target RNA (for tertiary complex formation) and measured total RNA in the Cas13d:RNA complexes by EMSA (Fig. 4f). We observed lower RNA intensity from OptoCas13d-off under blue light compared to the dark-incubated complex (Fig. 4f, g). Taken together, these results suggest that blue light does not affect the formation of binary crRNA:RfxCas13d complexes but does alter the binding of crRNA:RfxCas13d to its target.

Can we distinguish light-dependent effects on Cas13d complex formation versus catalysis in cells? To address this question, we reasoned that systems where a cellular response can be driven by a catalytically-inactive Cas13d (dCas13) offer the chance to observe light-dependent RNA binding in cells in the absence of Cas13d enzymatic activity. We tested two systems: a translational repression system in bacterial cells based on dCas13-based blockade of ribosome binding, and an RNA editing system in mammalian cells based on dCas13-ADAR protein fusion.

Taking inspiration from a recent study^31^, we designed a bacterial dCas13d system to bind a target mRNA and block translation. We transformed *E. coli* with two plasmids: one driving the expression of the *Ds*Red target and a *Ds*Red-targeting or non-targeting crRNA, and a second which expressed the desired dCas13d variant (dRfxCas13d, dOptoCas13d-off or dOptoCas13d-on) from either an arabinose-inducible promoter or a strong constitutive promoter (LacI^q^) (Fig. 4h). We induced Cas13d expression, exposed bacterial cultures to light or dark, and assessed the resulting *Ds*Red expression (Fig. 4i). We found that dRfxCas13d drove potent translational repression in the presence of a *Ds*Red-targeting crRNA, and that modest light-dependent changes in *Ds*Red could be observed for both dOptoCas13d-on and dOptoCas13d-off in the expected stimulus condition. We also observed some reduction in *Ds*Red expression in cells expressing a *Ds*Red-targeting crRNA compared to a non-targeting crRNA, even in the absence of dCas13d, likely indicative of antisense RNA base pairing and translational interference (Fig. 4i).

It was recently shown that a dead Cas13d variant could be fused to the ADAR2 base editor to perform RNA-guided base editing^32^. We thus reasoned that an ADAR2-fused dead OptoCas13d-off would exhibit light-dependent base editing only if light primarily acted on Cas13/mRNA complex formation. Alternatively, if light stimulation altered catalytic activity, base editing would be unaffected by optogenetic stimulation. To distinguish between these models, we generated catalytically inactive versions of wild-type RfxCas13d (dRfxCas13d) and its light-controlled variant (dOptoCas13d-off) by fusing them to the ADAR2-based RNA base editor, as described by Cox et al^5^. These constructs were evaluated using a dual-luciferase reporter system, in which *Cypridina* luciferase (CLuc) was used as the base editing target, and *Gaussia* luciferase (GLuc) served as an internal normalization control. The intended A-to-G conversion was designed to correct a premature stop codon at amino acid position 85 of the CLuc gene (Supplementary Fig. 7a)^5^. We only observed slight differences in base editing efficiency between dark and light conditions, with both exhibiting reduced editing activity compared to wild-type RfxCas13d (Supplementary Fig. 7b).

Taken together, biochemical and cellular assays provide evidence that our allosteric site in Cas13d partially couples to ternary complex formation. However, the modest effects stand in contrast to the potent changes in reporter expression observed in response to our catalytically active ChemoCas13d and OptoCas13d tools (Fig. 3). These data suggest that our stimulus-switchable Cas13d tools may rely on light-induced changes to both target RNA binding and cleavage for their high dynamic range.

### Development of ChemoCas13d for drug-inducible endogenous transcript knock-down

Our experiments so far suggest that our stimulus-switchable Cas13d tools (OptoCas13d-off, OptoCas13d-on and ChemoCas13d) perform especially well at controlled protein expression levels. We thus hypothesized that clonal mammalian cell lines, which typically maintain relatively tight distributions of engineered gene expression, could be derived with potent responses against both fluorescent reporters and endogenous target genes. To test this hypothesis, we generated and characterized a panel of 96 clonal ChemoCas13d cell lines to explore this system’s full potential for rapamycin-switchable RNA regulation.

**Fig. 5:**
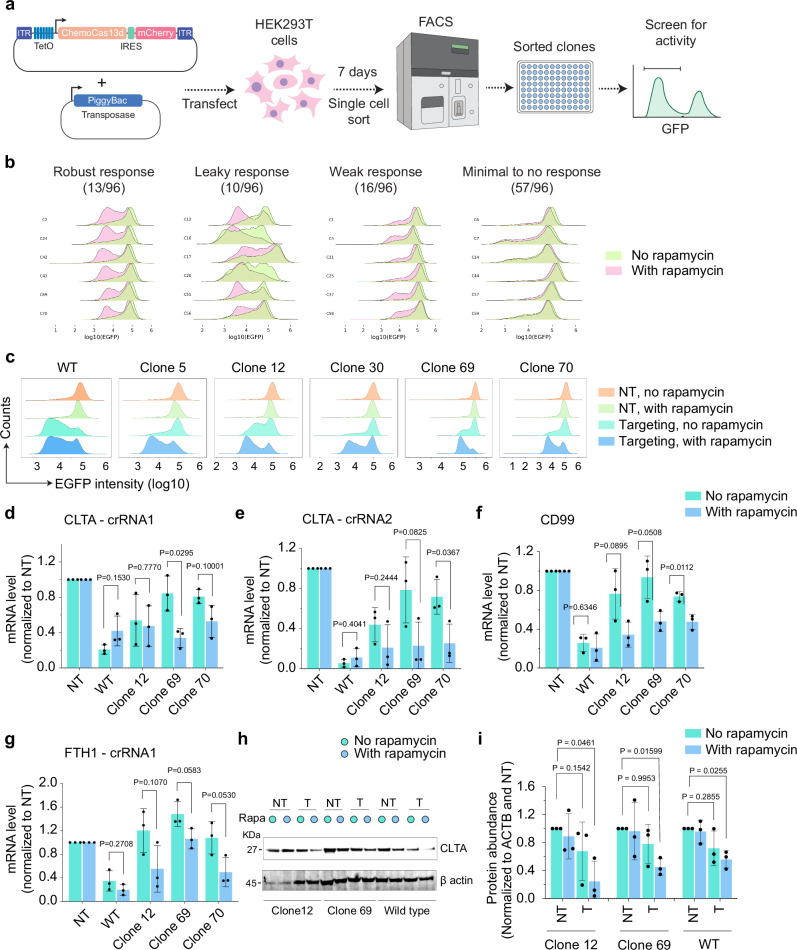
Generation and functional characterization of clonal ChemoCas13d cell lines. **a** Experimental schematic illustrating PiggyBac-mediated integration of a TetO-driven ChemoCas13d–IRES–mCherry cassette into HEK293T cells, followed by single-cell isolation by FACS to generate 96 clonal lines spanning a wide range of ChemoCas13d expression levels. **b** Primary functional screen of all 96 clones using an EGFP reporter assay. Representative flow cytometry histograms show EGFP fluorescence in the presence or absence of rapamycin, enabling classification of clones based on knockdown magnitude and inducibility. **c** Secondary characterization of selected high-performing clones using a non-targeting crRNA to assess basal versus rapamycin-induced activity, shown as EGFP fluorescence distributions under targeting and non-targeting conditions. **d–g** Endogenous mRNA knockdown analysis in representative inducible clones targeting CLTA, CD99, and FTH1. Transcript levels were quantified by RT–qPCR and normalized to non-targeting controls. **h** Western blot validation of rapamycin-dependent knockdown at the protein level in selected clones, showing reduced target protein abundance upon targeting and drug treatment relative to non-targeting and untreated controls. Uncropped gels are included as Supplementary Fig. S13. **i** Quantifications of protein abundance in h. Analysis was performed via ImageJ. For **c–i**, data are presented as mean ± S.D. of *n* = 3 biological replicates. Statistical significance was assessed using a two-tailed unpaired *t*-test. Source data are provided as a Source Data file.

We employed a doxycycline-inducible system for controlled Cas13d expression to mitigate any potential toxicity. The ChemoCas13d coding sequence, tagged with IRES-mCherry, was cloned downstream of a Tet-On promoter in a piggyBac vector (Fig. 5a). This construct was transfected into a 293 T landing pad cell line expressing a destabilized EGFP-PEST (293T-LP-dGFP)^22^ along with a PiggyBAC helper plasmid to facilitate genomic integration. Notably, 293T-LP-dGFP cells already stably express the reverse tetracycline-controlled transactivator (rtTA) from the landing pad locus, enabling transcriptional activation of RfxCas13d-LightR in response to doxycycline induction. We performed clonal selection following transfection and doxycycline treatment, obtaining clones from across a wide range of mCherry fluorescence. Because mCherry is expressed from the same transcript as ChemoCas13d, its fluorescence served as a quantitative proxy for ChemoCas13d expression across clones. This strategy allowed us to capture substantial heterogeneity in effector expression levels while maintaining an otherwise isogenic background.

We next functionally screened all 96 clones for RNA knockdown activity using an EGFP reporter assay (Fig. 5b). Each clone was transfected with an EGFP-targeting crRNA and analyzed by flow cytometry in the presence or absence of rapamycin to assess both basal and drug-induced ChemoCas13d activity. (Note that crRNA transfection is unlikely to target 100% of cells, so cell-to-cell variability in crRNA expression and reporter targeting is expected within each clone.) This screen revealed substantial variability in knockdown efficiency and inducibility between clones. The EGFP fluorescence distribution of each clone could be broadly classified into four phenotypic categories: clones exhibiting robust rapamycin-dependent knockdown, clones exhibiting leaky knockdown even in the absence of rapamycin, clones with weak knockdown, and clones showing minimal or no activity (Fig. 5b, Supplementary Fig. 8). These phenotypes were also correlated with overall ChemoCas13d expression in each clone (Supplementary Fig. 9). These results highlight the strong influence of genomic integration site and expression level on effective regulation of ChemoCas13d-mediated RNA targeting.

In addition to inter-clonal variability, we observed heterogeneity in some ChemoCas13d activity within individual clones that correlated with effector expression levels. When cells were analyzed without binning mCherry fluorescence level, several clones classified as weak or minimally responsive to doxycycline exhibited only modest EGFP knockdown at the population level (Supplementary Fig. 10a). However, gating specifically on high mCherry expression revealed markedly enhanced knockdown in that subpopulation, including in clones that otherwise appeared poorly responsive when analyzed in bulk (Supplementary Fig. 10b–d). At very high mCherry expression levels, we also detected lower EGFP expression even in the absence of rapamycin, indicative of leaky knockdown. Together, these findings highlight that both integration-dependent effects and cell-to-cell variability in doxycycline-induced expression contribute to the observed range of ChemoCas13d activity and underscore the importance of considering effector expression levels when evaluating inducible RNA-targeting systems.

From this primary screen, we selected 18 out of the 96 clones that displayed strong EGFP knockdown (including some clones with moderate leakiness) and further characterized their inducibility using a non-targeting crRNA control (Supplementary Fig. 11). This secondary screen was designed to decouple target-specific knockdown from nonspecific or background Cas13d activity. Most of these clones showed minimal basal effects in the absence of rapamycin or when transfected with a non-targeting crRNA, while maintaining strong on-target knockdown upon drug addition, consistent with tight chemical and RNA-based control (see e.g., Fig. 5c). Based on these criteria, we prioritized three representative clones (clones 12, 69, 70) with robust, rapamycin-dependent induction and varying degrees of background activity for detailed downstream analysis.

To determine whether inducible ChemoCas13d activity extended to endogenous transcripts, we performed mRNA knockdown experiments targeting multiple native genes using independent crRNAs (Fig. 5d–g, Supplementary Fig. 12). We selected *CLTA*, *FTH1*, and *CD99* as representative targets and quantified transcript levels by RT–qPCR in the presence or absence of rapamycin^33^. Across all three clones that were tested, targeting crRNAs induced substantial reductions in mRNA abundance following rapamycin treatment, whereas wild-type RfxCas13d showed knockdown regardless of rapamycin presence. Notably, clones 69 and 70, identified in the initial screen as exhibiting low leakiness, maintained low background knockdown in the absence of rapamycin under non-targeting conditions, indicating tight repression of ChemoCas13d activity. In contrast, clone 12, which displays higher mCherry fluorescence and thus elevated ChemoCas13d expression, exhibited moderate knockdown of some transcripts even in the absence of rapamycin (Fig. 5d, e, Supplementary Fig. 12a). This trend matching its behavior in the EGFP reporter knockdown assay (Fig. 5c), but it is important to note that we obtained noisier and more limited silencing in the endogenous gene case, possibly reflecting variability in knockdown efficiency due to target expression level, or measurement noise in the qPCR assay. Overall, these results indicate that basal Cas13d activity scales with effector expression level and underscore mCherry abundance as a key determinant of background RNA knockdown. Use of two independent crRNAs per target further demonstrated the robustness and reproducibility of ChemoCas13d-mediated knockdown across distinct crRNA sequences.

Finally, we validated that transcript-level repression resulted in corresponding reductions in protein abundance by western blot analysis (Fig. 5h, i, Supplementary Fig. S13). Consistent with the qPCR data, rapamycin treatment led to decreased CLTA protein levels in inducible clones, whereas untreated cells and wild-type controls showed no significant change. Together, these results establish a systematic pipeline for isolating clonal cell lines with tightly regulated ChemoCas13d activity and demonstrate that chemical induction enables efficient and specific RNA knockdown at both the mRNA and protein levels.

### Applications of OptoCas13d-on for light-inducible endogenous transcript knock-down

**Fig. 6:**
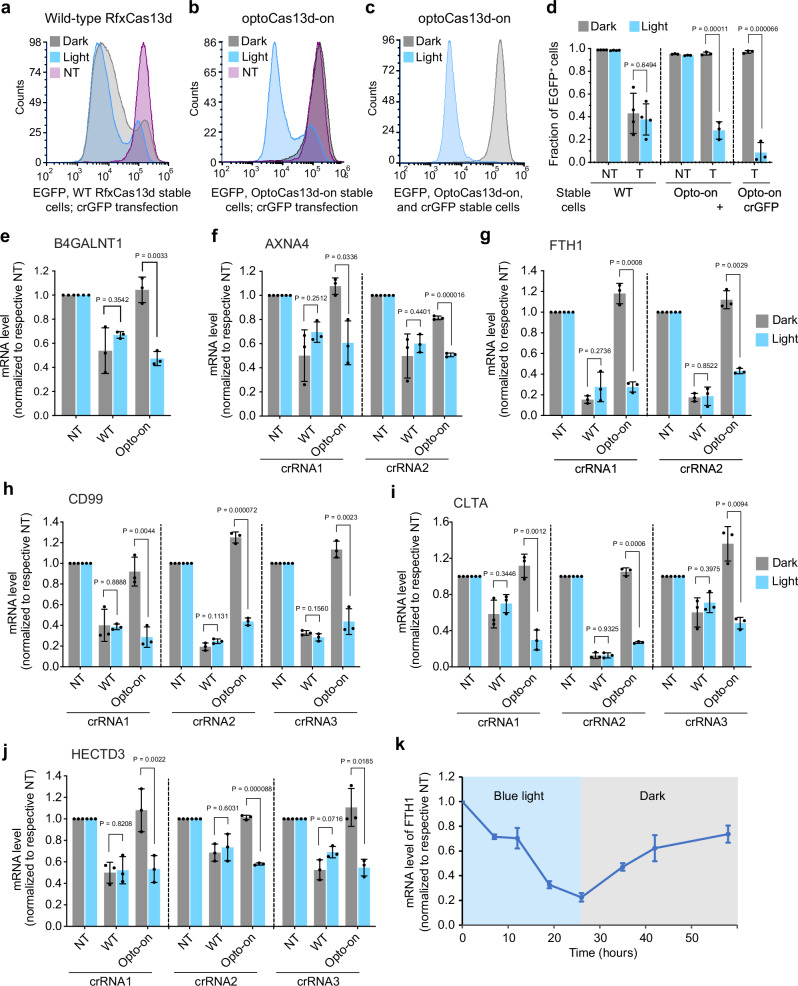
Endogenous transcript knockdown using OptoCas13d-on. **a**, **b** Flow cytometry histograms of EGFP fluorescence in stable cell lines harboring a constitutively-expressed EGFP reporter and doxycycline-inducible wild-type RfxCas13d (in (**a**)) or OptoCas13d-on (in (**b**)). Cells were transfected with an EGFP-targeting or non-targeting (NT) crRNA. For the targeting RNA, light and dark conditions are shown. **c** Flow cytometry histograms of EGFP fluorescence in a cell line stably expressing all three components: EGFP reporter, OptoCas13d-on, and anti-EGFP crRNA. Light and dark conditions are shown. **d** Quantification of the fraction of EGFP⁺ cells under the conditions described in (**a–c**). Points indicate data from *n* = 4, *n* = 3, and *n* = 3 biological replicates, respectively; mean ± S.D. are shown. Statistical significance was assessed using a two-tailed unpaired *t*-test. **e**-**j** qPCR quantification of the indicated mRNAs from a clonal OptoCas13d-on cell line that was transfected with crRNAs targeting the indicated mRNAs or non-targeting controls (NT). All results are normalized to respective non-targeting crRNA groups. **k** Time course experiment of OptoCas13d-on clonal cell line transfected with FTH1-targeting crRNA, followed by 26 h light duration and then 30 h dark duration. mRNA levels were measured at some time points. In panels **e-k**, data are presented as mean ± S.D. of *n* = 3 biological replicates. Statistical significance was assessed using a two-tailed unpaired *t*-test. Source data are provided as a Source Data file.

We also created a clonal OptoCas13d-on cell line for light-switchable regulation of both reporter genes and endogenous transcripts. This time, we performed clonal selection following transfection and doxycycline treatment, focusing on cells exhibiting mCherry fluorescence within the effective expression range (~10^4^ fluorescence units). We selected a suitable clone based on EGFP reporter knockdown after transfection with an EGFP-targeting crRNA. As expected, EGFP knockdown was observed exclusively under blue light exposure, with no detectable knockdown in the dark (Fig. 6a, b), demonstrating that OptoCas13d-on can act as a potent light-activated RNA expression switch. Stable integration of the EGFP-targeting crRNA via lentiviral transduction produced a fully homogeneous EGFP knockdown response to light (Fig. 6c), suggesting that an additional source of heterogeneity observed in our prior crRNA-transfected cell populations is driven by variable uptake and expression of the crRNA. To quantify the dynamic range and background activity of the system, we quantified the fraction of EGFP⁺ cells in WT and OptoCas13d stable lines following targeting or non-targeting crRNA delivery under dark or light conditions (Fig. 6d). OptoCas13d-on-mediated EGFP knockdown under light was statistically significant compared to dark conditions and non-targeting controls, whereas wild-type RfxCas13d mediated EGFP knockdown irrespective of illumination.

To further determine whether the OptoCas13d-on clonal line could be used to efficiently target endogenous target genes, we transfected it with a crRNA targeting the endogenous transcript *B4GALNT1*. Cells were either exposed to light for 5 h following transfection or maintained in darkness. After an additional 32 h, total RNA was extracted, and RT-qPCR was performed to quantify *B4GALNT1* mRNA levels (Fig. 6e). As expected, significant knockdown of *B4GALNT1* was observed only in light-exposed samples. We further evaluated the performance of OptoCas13d-on in silencing five additional endogenous transcripts by transfecting our cell line with multiple crRNAs targeting *AXNA4*, *FTH1*, *CD99*, *CLTA*, and *HECTD3* (Fig. 6f–j)^33^. In all cases, we observed robust light-inducible knockdown, demonstrating the versatility and efficacy of OptoCas13d-on for controlling endogenous gene expression.

Finally, we set out to determine the temporal dynamics and reversibility of light-induced knockdown after an acute change in illumination conditions. We conducted a time-course experiment using RfxCas13d-LightR transfected with an *FTH1*-targeting crRNA. Cells were illuminated for 26 h, after which they were transferred to dark conditions for an additional 25 h. mRNA levels were measured at multiple time points throughout the experiment (Fig. 6k). We observed a detectable reduction in *FTH1* mRNA levels within 12 h of light exposure, with a more pronounced knockdown as illumination duration increased, reaching a maximum effect between 18 and 26 h. Upon transitioning cells to dark conditions at the 26-h mark, *FTH1* transcript levels gradually recovered over time, demonstrating the reversibility of the system. These results confirm that RfxCas13d-LightR provides a tunable and reversible platform for the controlled knockdown of endogenous genes with high temporal precision.

## Discussion

We have identified a single site in RfxCas13d (QK634) that allows for light and chemical induction via domain insertion. This modularity allowed us to develop OptoCas13d-off (active in dark and inactive in light), OptoCas13d-on (active in light and inactive in dark), and ChemoCas13d (active in the presence of a chemical inducer). Recent studies have shown that toxicity associated with the promiscuous nuclease activity of Cas13d proteins can be mitigated by low effector and target RNA expression levels. Consistent with these findings, we observe selection against active Cas13d variants in our initial screen, and we find that light- and drug-switchable activity are optimal at specific RfxCas13d expression levels. Once appropriate expression levels are obtained, our ChemoCas13d and OptoCas13d systems exhibit high-quality stimulus-switchable control in stable cell line contexts. It should be noted that we generally obtained more reproducible and potent knockdown of endogenous genes with OptoCas13d-on compared to ChemoCas13, suggesting that there is likely to be room for further improvement in the rapamycin-controlled system.

We also performed in vitro biochemical assays to gain insight into the switchable mechanism of OptoCas13. We found that blue light illumination does not affect binding of OptoCas13d-off to its crRNA and that ternary complex formation between OptoCas13d-off, the crRNA, and target RNA is only partially (~40%) reduced. A similar two-fold reduction was observed in bacteria expressing catalytically dead OptoCas13d (dOptoCas13) in a translational repression assay, and only a minor change in RNA editing was observed in mammalian cells expressing an ADAR2-dOptoCas13d fusion. Together, these data are consistent with a modest but reproducible change in ternary complex formation upon optogenetic stimulation that fails to explain the strong effects that we observe on target RNA cleavage. Together, these data suggest that our stimulus-switchable Cas13d systems are likely to affect both complex formation and RNA nuclease activity.

The idea of optogenetically and chemically inducible Cas13d proteins has been explored by others^13–15^. Notably, these systems were based on inducible dimerization to reconstitute split PspCas13b or RfxCas13d proteins to restore their activity, and none report light-controlled RNA degradation comparable to that achieved by full-length Cas13d enzymes. While powerful, split systems can result in leaky activity in the absence of induced dimerization, as well as low enzyme activity after reconstitution. Our approach, identifying a domain insertion site for allosteric control, is quite effective in generating variants that exhibit either light or drug-induced enzymatic activity. This versatility provides end-users with the ability to knock down an RNA of interest either in the presence or absence of multiple stimulus modalities.

In the future, we envision that OptoCas13d could be used to control and perturb essential or tightly regulated cellular processes with high spatial and temporal precision, such as cell cycle progression, axonal RNA transport, and developmental gene expression. ChemoCas13d provides complementary capabilities: small molecule-based control over the initiation of programmed RNA degradation, albeit with limited reversibility and spatial precision. Such tools could also be used to study biological processes, such as investigating sub-populations of infected cells during viral-host interactions. The emergence of single-cell RNA sequencing has demonstrated that in many tissues and organisms, there is a diverse array of cell types, each with different gene regulatory networks. Our tools complement these sequencing efforts by enabling an end user to perturb RNA levels in a precisely defined subset of cells both in time and in space.

## Methods

### Plasmid construction

Plasmids associated with Mu transposition were originally obtained as a gift from David Savage, which include pUCKanR-Mu-BsaI (Addgene #79769) and pATT-Dest (Addgene #79770)^25^. Coding sequence of RfxCas13d and expression vectors of RfxCas13d and crRNA were also obtained as a gift from Patrick Hsu, which include pXR001: EF1a-CasRx-2A-EGFP (Addgene #109049) and pXR003: CasRx crRNA (Addgene #109053)^4^. pLZD047 was constructed by cloning the RfxCas13d coding sequence from pXR001 into pATT-Dest with BsmBI restriction sites added on both sides of RfxCas13d through in-fusion assembly (Takara #638911). Landing pad plasmids, including recombination plasmids pJG_082 AttB_mCherry_Bgl, pJG_083 AttB_EGFP_Bgl2 and the pJG_075 Bxb1, which expresses Integrase essential for gene integration, were obtained as gifts from Jacob Goell (Rice University, Hilton Lab). pLZD081 was constructed by cloning NLS-RfxCas13d-NLS into attB recombination plasmid through in-fusion assembly, and IRES-mCherry, the PCR product with pIRES2-mCherry-p53 deltaN (Addgene #49243) as template was tagged to NLS-RfxCas13d-NLS also through in-fusion assembly^34^. For transient transfection, Cas13d and its variants were cloned into the PQ vector through in-fusion assembly, and crRNAs were cloned into pXR003. For stable expression, Cas13d and its variants tagged with IRES-mCherry were cloned into Piggybac vector downstream Tet-on promoter, and crRNAs were cloned into LRG2.1 vector, which contains a constitutively expressed iRFP as the selection marker. LRG2.1 vector, which was originally LRG2.1-TagBFP2 (Addgene #124773), was obtained as a gift from Jason Sheltzer^35^. The AsLOV2 (408-543) sequence used in this study is the same as we previously used^26^. LightR sequence was cloned from LightR-bRaf-mVenus (Addgene #162154)^20^. UniRapR sequence was cloned from pUSE-Src-YF-UniRapR-mCerulean-myc (Addgene #45381)^21^. See Supplementary Table 1 for a list of plasmids used in this study.

### Construction of AsLOV2 insertion library

To obtain a transposon that contains a chloramphenicol resistance gene, PCR was performed with pUCKanR-Mu-BsaI as template, 5′-taggcaccccaggctttacac-3′ as forward primer and 5′-tctgtaagcggatgccggga-3′ as reverse primer. The product was purified and digested with HindIII and BglII, followed by purification with NucleoSpin Gel and PCR Clean-up Columns (Takara, #740609). The resulting DNA was directly used as a transposon. Transposition reactions were conducted in a total volume of 20 μL with the following components: 0.14 pmol pLZD047, 0.55 pmol transposon DNA, 4 μL 5 × MuA reaction buffer and 1 μL 0.22 μg/μL MuA transposase enzyme (Thermo Fisher Scientific, #F750). Reactions were incubated for 18 h at 30 °C followed by 10 min at 75 °C to heat inactivate MuA transposase. Completed reactions were cleaned up with DNA Clean & Concentrator-5 Kit (Zymo Research, #D4014) and eluted with 20 μL nuclease-free water (Thermo Fisher Scientific, #AM9932), which was then transformed into 200 μL TransforMax Electrocompetent *E. coli* (Lucigen, #EC10010). An aliquot of the recovery culture was spread on an LB agar plate with carbenicillin (Gold Biotechnology, #C-103-5) and chloramphenicol (Gold Biotechnology, #C-105-5) antibiotics to assess reaction efficiency. The remaining recovery culture was transferred to 50 ml LB with chloramphenicol and carbenicillin to select for plasmids with transposon insertion, and after overnight growth, the library was collected with the Plasmid Plus Midi Kit (Qiagen, #12943), which was named pLZD047_CmR01. Qubit dsDNA Quantification Assay Kits (ThermoFisher Scientific, #Q32851) were used to quantify library DNA concentration in all the following steps.

To isolate library members containing RfxCas13d with CmR insertion, BsmBI digestion of pLZD047_CmR01 was performed. Total amount of 5 µg pLZD047_CmR01, 25 µL 10 × NEBuffer 3.1, 50 units BsmBI_v2 (New England Biolabs, #R0739S) in a total volume of 250 µL was mixed and incubated at 55 °C for 1 h followed by 80 °C for 20 min. Gel separation was performed afterward, and the band with the expected size of RfxCas13d with CmR insertion (4154 base pairs) was cut and purified with Zymoclean Gel DNA Recovery Kit (Zymo Research, #D4007), which was named pLZD047_CmR01_DNA. Next, to clone pLZD047_CmR01_DNA back to the original pLZD047 vector, backbone PCR was performed with pLZD047 as the template and primers as follows: Forward primer: tttttcggtctccGGATCgagacggttaagatc Reverse primer: tttttcggtctccGCTGGgagacgatggtatatctccttcttaaagttaaaca.

The PCR product was then digested with BsaI-HF_v2 (New England Biolabs, #R3733S), which created sticky ends on both sides that match up with the pLZD047_CmR01_DNA, and the digested product was termed pLZD047_DNA. T4 DNA ligase (New England Biolabs, #M0202S) was then used to ligate pLZD047_DNA with pLZD047_CmR01_DNA following the product protocol (https://www.neb.com/en-us/protocols/0001/01/01/dna-ligation-with-t4-dna-ligase-m0202), and the resulting library was termed pLZD047_CmR02.

Next, Golden Gate cloning was used to replace the chloramphenicol resistance gene with the AsLOV2 domain in pLZD047_CmR02. In detail, pLZ047_CmR02 was used as the backbone, and the insert was linear DNA with AsLOV2 (408–543) coding sequence and two BsaI digestion sites on both ends. Briefly, 75fmol of backbone and 210 fmol of AsLOV2 insert were mixed with 37.5 units BsaI_HFv2 (New England Biolabs, #R3733S), 2000 units T4 DNA Ligase (New England Biolabs, #M0202S) and 5 μL 10 × T4 DNA Ligase Reaction Buffer in a total volume of 50 μL. The reaction was incubated 2 min at 37 °C, 5 min at 16 °C (first two steps cycled 50 times), 20 min at 60 °C and 20 min at 80 °C. Reactions were purified with DNA Clean & Concentrator-5 Kit and transformed into 100 μL TransforMax Electrocompetent *E. coli*. Cells were then transferred to 25 ml LB with carbenicillin, and the library was collected with the Plasmid Plus Midi Kit, which was named pLZD047_LOV. Another step of BsmBI digestion was performed for pLZD047_LOV, and the band with a size corresponding to RfxCas13d-LOV (3320 base pairs) was purified, with the name pLZD047_LOV_DNA

Lastly, Golden Gate cloning was used again to clone pLZD047_LOV_DNA into a landing pad recombination plasmid. PCR was performed with the pLZD81 as the template, forward primer taccatcgtcTCCGGATCCggacctaagaaaaagaggaaggtggcg and reverse primer cttaaccgtctcaGCTGGCCTCCACCTTtCTC to obtain the linearized landing pad recombination plasmid. For golden gate cloning, pLZD047_LOV_DNA was used as the insert, and the linearized landing pad recombination plasmid was used as the backbone. Briefly, 58 fmol backbone and 78 fmol of insert were mixed with 15 units BsmBI_v2, 800 units T4 DNA Ligase and 2 μL 10 × T4 DNA Ligase Reaction Buffer in a total volume of 20 μL. The reaction was incubated 2 min at 42 °C, 5 min at 16 °C (first two steps cycled 50 times), 20 min at 60 °C and 20 min at 80 °C. Reactions were purified using a DNA Clean & Concentrator-5 Kit and transformed into 100 μL TransforMax Electrocompetent *E. coli*. Cells were then transferred to 30 ml LB with carbenicillin, and the library was collected with the Plasmid Plus Midi Kit, which was named pLZD081_LOV.

### Construction of the reporter cell line for selections and transient transfection assay

HEK293T landing pad (293T-LP) cell line was a gift from Kenneth Matreyek (Case Western Reserve University), which was derived from HEK293T that had been bought from ThermoFisher. HEK293T landing pad cell line with constitutively-expressed eGFP (293T-LP-dGFP) was constructed by incorporating constitutively-expressed eGFP in pHR vector (pLZD075) into 293T-LP genome through lentivirus transduction. All the cells were kept in DMEM (ThermoFisher, #11995073) supplemented with 10% FBS (R&D Systems, #S11150), 1% Pen Strip (Gibco, #15140-122) and 2 mM L-Glutamine (Gibco, #25030-081) throughout all experiments.

To produce lentiviral particles, HEK293T cells were plated on a 6-well plate and grown up to 40% confluency. At that point, they were co-transfected with pLZD075 and lentiviral packaging plasmids (pMD and CMV) with FuGENE HD (Promega, #E2311). Specifically, 1500 ng pLZD075, 1330 ng pCMVdR8.91 and 170 ng pMD2.G mixed with 9 μL Fugene HD transfection reagent were used for each well in a 6-well plate. Virus was collected after 53 h, filtered using a 0.45 mm filter. Polybrene (Sigma-Aldrich, #TR-1003-G) was added to the viral particles to a final concentration of 4 μg/mL. 293 T LP cell line was plated on a 6-well plate and infected with 350 μL of the virus at 40% confluency, and EGFP-positive clonal cells were sorted 52 h post the infection time. Two weeks after clonal sorting, several clones were characterized through flow cytometry and one clone showing uniform EGFP expression was chosen as 293T-LP-dGFP for further incorporation of Cas13d variants or crRNA, or for transient transfection assays.

Next, EGFP-targeting crRNA (or non-targeting crRNA as a control) in LRG2.1 vector (pLZD078 for EGFP-targeting crRNA, and pLZD079 for non-targeting control crRNA) was further incorporated into 293T-LP-dGFP through lentivirus transduction, with a constitutively-expressed iRFP as the selection marker. To produce lentiviral particles, HEK 293 T cells were plated on a 6-well plate and grown up to 40% confluency. At that point, they were co-transfected with pLZD078 or pLZD079 and lentiviral packaging plasmids (pMD and psPAX2.0) with FuGENE HD (Promega, #E2311). Specifically, 1500 ng pLZD078, 1850 ng pCMVdR8.91 and 300 ng pMD2.G mixed with 10.95 μL Fugene HD transfection reagent were used for each well in a 6-well plate. Virus was collected after 50 h, filtered using a 0.45 mm filter. Polybrene (Sigma-Aldrich, #TR-1003-G) was added to the viral particles to a final concentration of 4 μg/mL. 293 T LP cell line was plated on a 6-well plate and infected with 300 μL of the virus at 40% confluency, and the iRFP clonal cell was sorted 48 h post the infection time. Two weeks after clonal sorting, several clones were characterized through flow cytometry and one clone showing uniform iRFP expression was chosen as the reporter cell line for the following negative selection experiments, termed 293T-LP-dGFP-GFP_crRNA or 293T-LP-dGFP-NT_crRNA.

### Integration of Cas13-LOV insertion library into 293 T landing pad reporter cell line and follow-up negative selections

HEK293T-LP-dGFP_crRNA cell lines were plated on 6-well plates one day before transfection. Recombination was performed by transfecting cells with 1500 ng of pJG75 Bxb1 and 2500 ng of AsLOV2 insertion library pLZD081_LOV in doxycycline-free media with 9 μL FuGENE HD transfection reagent. 32 h following transfection, the media was changed to media supplemented with 2 μg/mL doxycycline. 62 h after media replacement, cells that are mCherry positive and BFP negative were sorted to a new 6-well plate with flow cytometry. Before flow cytometry, cells were detached with trypsin and resuspended in DMEM containing 10% serum. Flow cytometry was performed with SH800S Cell Sorter equipped with Sony 100 μm Sorting Chip (Sony Biotechnology, LE-C3210). BFP was excited with a 405 nm laser, and the emitted light was collected after passing through 450/50 nm band pass filters. EGFP was excited with a 488 nm laser, and the emitted light was collected after passing through 525/50 nm bandpass filters. mCherry was excited with a 561 nm laser, and the emitted light was collected after passing through 665/30 nm band pass filters. Manual compensation was performed to decouple crossovers among BFP, EGFP and mCherry signals. Before sorting, live, single cells were gated using FSC-A and SSC-A (for live cells) and FSC-A and FSC-H (for single cells), and at least 100,000 cells were sorted and plated on a new 6-well plate in doxycycline-free media. Another round of doxycycline induction and sorting was performed two weeks after the first sorting to further enrich mCherry-positive and BFP-negative cells.

### Extraction of genomic DNA and next-generation sequencing

PureLink Genomic DNA Mini kit (Invitrogen, #K182001) was used to extract genomes from library cells following the protocol in the manual, and PCR was performed with forward primer 5′-CCAGGGCTCGAGACCGCAACTACACGCCACC-3′ and reverse primer 5′-AGCTTCGAATTCGGGGCGGATCAGCTTGGTAC-3′ to amplify the library fragments from the genome. Library fragment DNA was then sheared with a Covaris S220 focused ultrasonicator using AFA microTUBEs (Covaris, #PN 520052) to an approximate size of 300–400 base pairs. NEBNext® Ultra™ II DNA Library Prep with Sample Purification Beads (New England Biolabs, #E7103S) was used to prepare a DNA library for sequencing from sheared DNA. Sheared DNA and prepared samples were analyzed for size distribution on an Agilent 2100 Bioanalyzer using DNA 1000 chips (Agilent Technologies). Double-stranded DNA concentrations of the adapter-prepared samples were measured with a dsDNA HS Assay Kit (Invitrogen, #Q32851) on a Qubit Fluorometer. A normalized pool of samples was run on a MiSeq Nano 300 nt or MiSeq Micro 300 nt for over 300 cycles. Analysis of FASTQ files of sequencing results was performed through MATLAB R2021b. For deeper sequencing to map all of the initial integration sites in Fig. 1D, Nanopore sequencing of the same initial libraries was carried out by Plasmidsaurus and the AsLOV2 insertion sites into Cas13d were mapped using custom Rust code. All code used to map reads is available on the Toettcher laboratory’s GitHub page: https://github.com/toettchlab/Zhu-Nguyen2025.

### Cell transient transfection for EGFP knockdown assay

In all Cas13d variants, transient transfection for EGFP-knock down assays, plasmid encoding Cas13d variant and plasmid encoding crRNA were double-transfected into 293T-LP-dGFP. For transfection, the Cas13d variant coding sequence is cloned to the PQ vector, and EGFP targeting crRNA (GFP_crRNA) or non-targeting control crRNA (NT_crRNA) was cloned into the pXR003 vector. 293T-LP-dGFP cells were plated on 12-well plates 1 day prior to transfection. 400 ng of Cas13d plasmid and 400 ng of crRNA plasmid were mixed with 2.4 μL FuGENE HD Transfection Reagent and transfected into 293 T-LP-dGFP. For the light-based assay, all the cells were kept in the dark for 5 h post-transfection and then irradiated with 450 nm blue LEDs at an intensity of 0.80 mW/cm^2^ or remained in the dark for an additional 26 h before characterization with flow cytometry. For rapamycin-based assay, 1 h prior to transfection, the cell media was replaced with fresh media with 0.01 μM rapamycin (Sigma-Aldrich, #553211), and flow cytometry was performed 28 h after transfection.

### Protein expression and purification

Protein expression plasmid was constructed by cloning a mammalian codon-optimized CasRx gene (gift from Patrick Hsu, Addgene #109049 and #109050 for catalytically active and inactive variants) into a bacterial expression vector bearing 6xHis and TEV cleavage sites (gift from Scott Gradia, Addgene #29653). The AsLOV2 domain was then inserted into this expression backbone using Infusion cloning.

For protein expression, plasmids were transformed into Rosetta™ 2(DE3) Singles™ competent cells following the manufacturer’s instructions. Individual colonies were picked the next day and inoculated in 25–30 mL LB broth for 14–18 h. The culture was then scaled up to 2 L with Terrific Broth and continued to be shaken at 37 °C until OD600 = 0.8–1.0. The culture was next placed on ice for 30–45 min followed by the addition of 1 mM IPTG to induce CasRx expression. The culture was transferred to a shaker and incubated at 18 °C for 14–18 h.

For protein purification, cell pellets were harvested via centrifugation. The pellets were then resuspended in lysis buffer (500 mM NaCl, 50 mM Tris-HCl, pH = 7.5, 20 mM imidazole, 1 mM TCEP-HCl, 0.5 mg/mL lysozyme, 0.5 mM PMSF, and 0.1 mg/mL DNase I) and subjected to sonication. Cell lysate was then centrifuged at 40,000 × *g* for 30 min followed by filtering through a 0.22 um syringe filter. The clarified lysate was injected into Nuvia IMAC, Ni-charged (Biorad) pre-equilibrated with buffer A (500 mM NaCl, 50 mM Tris-HCl, pH = 7.5, 20 mM imidazole, 1 mM TCEP-HCl) via the NGC Quest 10 Plus FPLC system (Biorad, #12009287). In the case of OptoCas and dOptoCas, 5 mM Flavin Mononucleotide was added to buffer A prior to purification. The column was eluted using buffer B (500 mM NaCl, 50 mM Tris-HCl, pH = 7.5, 300 mM imidazole, 1 mM TCEP-HCl), pooled together and dialyzed against buffer C (250 mM NaCl, 50 mM Tris-HCl, pH = 7.5, 300 mM imidazole, 1 mM TCEP-HCl, 10% glycerol) overnight. The next day, the protein mixture was concentrated and injected into the cation exchange column Macro-Prep High S (Biorad, #12009272) using the same FPLC system. The eluted fractions were collected by gradually exchanging buffer C with buffer D (2 M NaCl, 50 mM Tris-HCl, pH = 7.5, 300 mM imidazole, 1 mM TCEP-HCl, 10% glycerol) in a gradient fashion. Protein purity was analyzed using gel electrophoresis, pooled together, dialyzed against the final buffer (600 mM NaCl, 50 mM Tris-HCl, pH = 7.5, 300 mM imidazole, 2 mM DTT, 10% glycerol), flash frozen and stored at −80 °C until use.

### AlphaFold 3

Structural models of wild-type RfxCas13d (WT), optoCas13d-off, optoCas13d-on, and chemoCas13d were predicted using AlphaFold 3 (AF3) via the DeepMind online server (https://deepmind.google/science/alphafold/). For each variant, both binary complexes and ternary complexes incorporating regulatory or effector domains were modeled. Protein and RNA sequences were derived from experimental constructs and validated target sites, with effector domains appended using designed linkers where applicable. Complexes were modeled using AF3 multimer mode with default parameters and automated multiple sequence alignment generation. For each condition, five independent models were generated and ranked by predicted local distance difference test (pLDDT), and the highest-confidence model was selected for analysis. Predicted structures were analyzed using ChimeraX to assess conformational differences in RNA-binding interfaces and catalytic regions.

### In vitro trans-cleavage assay

To quantify Cas13d trans-cleavage activity, we used a fluorescence-based reporter assay in which cleavage of a short, quenched RNA substrate results in increased fluorescence. In this assay, target RNA recognition by Cas13d induces a conformational activation of the HEPN nuclease domains, which enables non-specific cleavage of the reporter RNA in trans. Therefore, fluorescence intensity serves as a proxy for the enzymatic activity of Cas13d following guide–target pairing. Because both cis-cleavage of the target RNA and trans-cleavage of collateral substrates require the same activated nuclease state, changes in trans-cleavage activity under dark and light conditions reflect differences in Cas13d activation upon target engagement. While this assay does not directly measure target RNA cleavage, it provides a sensitive and quantitative readout of Cas13d nuclease activation^36^.

Cleavage assays were adapted from previously described protocols30. A master mix was created with 1 × reaction buffer (20 mM HEPES pH 8.0 with 60 mM KCl and 3.5% PEG-8000), 10 mM MgOAc, 1.33 U µl−1 murine RNase inhibitor (New England Biolabs), 6.25 μM of crRNA (IDT), and 5 μM of either WT Cas13d or Cas13d containing AsLOV2. The crRNA and Cas13d were incubated at 37 °C for 15 min to complex under blue light or in the dark. Subsequently, 50 ng of target RNA was added to the reaction and incubated at 37 °C for another 15 min. Water was used in replacement of a target for no target controls (NTCs). Subsequently, 133.33 nM polyU (6 uracils; IDT)) Quenched HEX reporter was added to each reaction. 15 μL reactions were loaded in technical triplicate onto a Greiner 384 well clear-bottom microplate (item no. 788096). The reactions were incubated at 37 °C for up to 3 h with fluorescent readings taken every 5 min using an Agilent BioTek Cytation 5 microplate reader (excitation: 485 nm, emission: 525 nm).

### Electrophoretic mobility shift assay (EMSA)

For in vitro gel shift assays to visualize binary complex formation, 20 μL samples were prepared to reach final concentrations of 500 nM 3’FAM-GFP targeting-crRNA and either 400 nM wild-type CasRx or 400 nM CasRx-AsLOV2 in 1 × binding buffer (24 mM KCl, 4 mM Tris-HCl, pH 8.0, 0.4 mM DTT, 10% glycerol, 0.1 mg/mL BSA, 5 mM MgCl_2_). For samples meant to visualize ternary complex formation, the inactive versions of these enzymes were used, and 80 nM of a Cy5-labeled 107-nucleotide EGFP target RNA was also included. After preparation, samples were allowed to complex either in the dark or under blue light for 15 min at 37 °C before being mixed with gel loading dye and run on 5% polyacrylamide gels (Biorad, #4565013) in 0.5 × TBE buffer. Gel boxes were placed on ice and kept under aluminum foil to prevent light interference following complexing. crRNA signal was visualized by imaging the FAM channel on an Azure Biosystems 600 imager. Gels were then stained with SYBR gold (Invitrogen, #S11494) and re-imaged using the SYBR gold channel in order to visualize both crRNA and target RNA.

### Generation of plasmids for bacterial assays

A pCDF-pBAD plasmid was used as a backbone for the cloning of all CasRx constructs in bacterial assays. RfxCas13d was cloned from pET28b-RfxCas13d-His6 (Addgene #141322)^37^ into pCDF-pBAD using Gibson Assembly (5x ISO buffer: 25% PEG-8000, 500 mM Tris-HCl pH 7.5, 50 mM MgCl_2_, 50 mM DTT, 4 mM dNTP mix, 5 mM NAD; T5 exonuclease, Epicenter; Phusion DNA polymerase, New England Biolabs; Taq DNA ligase, New England Biolabs). The catalytically dead dRfxCas13d was generated by site-directed mutagenesis (Phusion High-Fidelity DNA-Polymerase, ThermoFisher). LOV2-dCasRx was generated by Gibson Assembly, with the pET19b-SUMO-*As*LOV2 plasmid^38^ as template for the insert. LightR-dCasRx was generated by Gibson Assembly, with the *E. coli* codon-optimized LightR sequence (synthesized by GeneArt, Regensburg) as template for the insert.

pET28c-*Ds*RedExpress2^39^ was used as a reporter plasmid. The crRNA expression cassette was introduced using extension PCR (Phusion High-Fidelity DNA-Polymerase, ThermoFisher) and subsequent blunt-end ligation (PNK, PEG4000, and T4 ligase, ThermoFisher).

### Opto-dRfxCas13d reporter gene assay in bacteria

pCDF-pBAD Opto-dCasRx constructs were transformed into chem. comp. *E. coli* CmpX13^40^ carrying pET28c-*Ds*RedExpress2-crRNA and plated on LB agar plates containing streptomycin and kanamycin. On a 96-well microtiter plate (96 F clear plate, ThermoFisher) 200 µL LB with streptomycin and kanamycin was inoculated with a single colony, the plate sealed with a breathable film (BF-410400-S, Corning) and incubated at 30 °C for 24 h with agitation. 1 µL of the starter culture was then spotted onto an inducing LB agar plate containing streptomycin, kanamycin, 200 µM IPTG, and 6 mM arabinose. The plates were incubated under darkness or blue light ( ~ 60 µW/cm^2^ at 450 nm) at 37 °C for 18 h. Spotted colonies were then resuspended in 200 µL H_2_O in a 96-well clear plate, and A_600_ was recorded using a plate reader (Tecan Infinite M200 Pro). Measured suspension was diluted 1:5 in 200 µL H_2_O in a 96-well black plate, and the *Ds*Red fluorescence was recorded (ex: 554 ± 9 nm; em: 591 ± 20 nm).

### Integration of Cas13d variants into 293T-LP-dGFP with Piggybac transposition

RfxCas13d variants coding sequences tagged with IRES-mCherry were first cloned to the Piggybac vector downstream of the tetracycline (Tet) inducible promoter. One example is pLZD239, which contains Cas13LightRQK634-IRES-mCherry. rtTA (reverse tetracycline-controlled transactivator) was already stably expressed in 293T-LP-dGFP cell lines. To incorporate pLZD239 into 293T-LP-dGFP (or 293T-LP-dGFP-GFP_crRNA with the same protocol), 2000ng pLZD239, 500 ng Piggybac helper plasmid was mixed with 18 μL FuGENE HD Transfection Reagent and transfected into 293T-LP-dGFP. The cells were maintained in doxycycline-free media unless otherwise mentioned. Six days after transfection, the media were changed to media supplemented with 2 μg/mL doxycycline. After another 56 h, flow cytometry was performed, and mCherry-positive cells were bulk sorted. One week afterward, another round of flow cytometry was performed, and individual mCherry-positive cells were sorted into each well of a 96-well plate. The resulting clonal cell line was termed 293T-LP-dGFP-Cas13LightR or 293T-LP-dGFP-GFP_crRNA-Cas13LightR.

### Base editing of luciferase reporter

Dual secreted luciferase reporter (EF1alpha-Gaussia luciferase, CMV-Cypridina luciferase) was obtained as a gift from Feng Zhang (Addgene plasmid # 181934) and modified to install a premature stop codon at amino acid 85 of the CLuc gene. 18–24 h prior to transfection, 10,000 HEK293T cells were seeded in two 96-well plates. Next, 40 ng of dual luciferase reporter plasmid, 150 ng of wild-type dRfxCas13d-ADAR or dOptoCas13d-QK634-AsLOV2-ADAR protein, and 300 ng of crRNA targeting cLuc were mixed with Lipofectamine 3000 and P3000 reagent (Invitrogen, #L3000015) and co-transfected into each well. Four hours later, one of the 96-well plates was then treated under blue light for 48 h. Medium containing luciferase in both light and dark-treated plates was used for luciferase analysis using UltraBriteTM Cypridina-Gaussia dual luciferase assay reagent (Targeting Systems, #DLAR-4 SG-1000) and Biotek Cytation 5 plate reader with automatic gain and 1 s exposure time (Agilent). Due to constitutive expression of GLuc, its signal was adjusted 100-fold lower than the actual measurements prior to background normalization with CLuc signal.

### Cell transient transfection for knocking down endogenous transcripts

For transfection, crRNA targeting endogenous transcripts or non-targeting control crRNA (NT_crRNA) was cloned into the pXR003 vector. 293T-LP-dGFP-Cas13LightR was used, which contains doxycycline-inducible Cas13LightRQK634. Cells were plated on 12-well plates in 2 μg/mL doxycycline media 1 day prior to transfection. During transfection, 800 ng of crRNA plasmid were mixed with 2.4 μL FuGENE HD Transfection Reagent and transfected into 293 T-LP-293 T-LP-dGFP-Cas13LightR. All the cells were kept in the dark for 5 h post-transfection and then irradiated with 450 nm 5 mm blue LEDs (Digikey, Inc) at an intensity of 0.80 mW/cm^2^ or remained in the dark for an additional 32 h before follow-up RT-qPCR experiments.

### RT-qPCR

To analyze the relative expression of endogenous transcripts, total RNA was extracted from cells with the RNeasy Mini Kit (Qiagen #74106) following the protocol in the manual. Reverse transcription was then performed with LunaScript RT SuperMix Kit (NEB, #E3010), and the products were directly mixed with qPCR primers and Luna Universal qPCR Master Mix (NEB #M3003L) for qPCR assay. The experiment follows the protocol for Two-step RT-qPCR using the LunaScript RT SuperMix Kit (NEB, #E3010) and the Luna Universal qPCR Master Mix (NEB, #M3003) available on the NEB website. Sequences of crRNA and qPCR primers are provided in Supplementary Tables 2, 3.

### Western blotting

HEK293T cells constitutively expressing ChemoCas13d were plated in 24-well plates 18–24 h prior to transfection in medium supplemented with 2 μg/mL doxycycline. Cells were transfected with crRNA the following day, followed by the addition of 0.01 nM rapamycin 4 h post-transfection. Cells were then incubated at 37 °C with 5% CO₂ for an additional 24–32 h.

Cells were lysed in 150 μL mammalian cell lysis buffer (GoldBio, GB-180-100) and clarified by centrifugation at 16,000 × *g* for 15 min at 4 °C. Clarified lysates were resolved by PAGE (GenScript, M00657), transferred to a blotting membrane (Thermo Fisher Scientific, IB301001) using the iBlot 2 transfer system, and incubated with primary in 1:1000 dilution (Proteintech, 10852-1-AP and Cell Signaling Technology, 3700S) and secondary antibodies in 1:5000 dilution overnight (Licor, 926-32211 and 926-68070). Blots were imaged and analyzed using a LI-COR Odyssey CLx imager.

### Statistics and reproducibility

All cell culture experiments were performed in at least three independent biological replicates. No data were excluded from the final analyses, and no statistical methods were used to predetermine sample size. The reproducibility of experimental findings was verified across multiple independent sessions with consistent results. Statistical significance was determined using a two-tailed unpaired *t*-test, with *P* < 0.05 considered statistically significant. All data are presented as mean ± S.D. unless otherwise indicated.

### Reporting summary

Further information on research design is available in the Nature Portfolio Reporting Summary linked to this article.

## Supplementary information

Supplementary Information

Reporting Summary

Transparent Peer Review file

## Source data

Source Data

## Data Availability

There are no restrictions on data availability. DNA sequencing files have been deposited in the NIH Sequence Read Archive, accession code PRJNA1260547. All other data analyzed in the paper are supplied as Source Data. Plasmids for OptoCas13d-on and ChemoCas13d have been deposited in Addgene (Plasmid # 248105 and # 248111, respectively). All other plasmids and cell lines will be shared upon request from Jared Toettcher (toettcher@princeton.edu) within 1 month. Source data are provided with this paper.

## Code availability

The code for analyzing next-generation sequencing data is provided in the paper’s GitHub repository [https://github.com/toettchlab/Zhu-Nguyen2025].
