# Supplementary material for: Multimodal control of Cas13d activity through domain insertion at an allosteric hotspot
Source: Nat Commun. 2026 Jun 3;17:7146. doi: 10.1038/s41467-026-73645-5 (PMC13396516; doi:10.1038/s41467-026-73645-5)
Supplement: Supplementary file 1 — Supplementary Information [file 41467_2026_73645_MOESM1_ESM.pdf]

Supplementary Information

Multimodal control of Cas13 activity through domain insertion at an allosteric hotspot

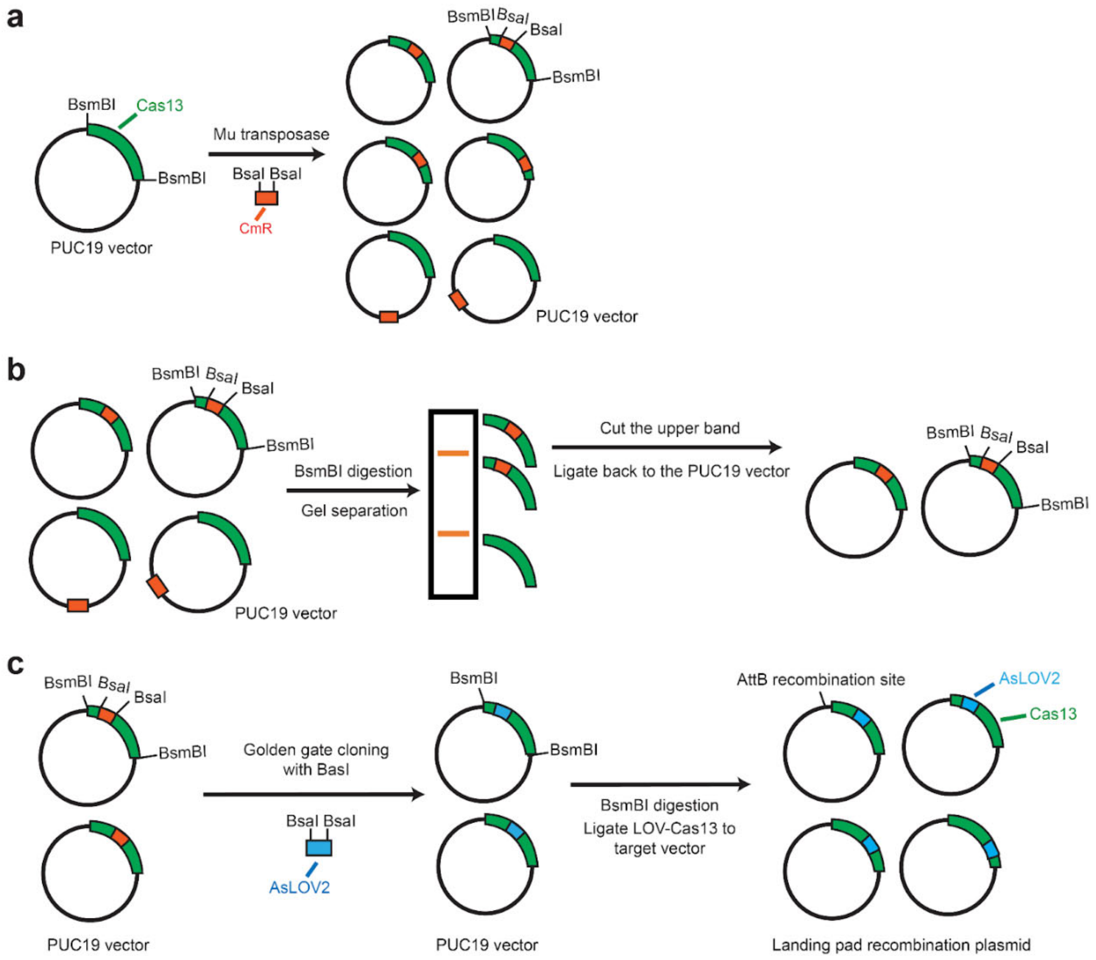

Supplementary Figure 1. Schematic of generation of OptoCas13d variant library. (a) Transposition reaction to randomly insert chloramphenicol-resistant genes (CmR) into a pUC19 vector containing RfxCas13d coding sequences. **(b)** BsmBI digestion and gel separation to purify fragments of RfxCas13d with CmR insertion and ligate them back to the original PUC19 vector with two BsmBI restriction sites on both sides of RfxCas13d sequence present. **(c)** BsaI-based golden gate cloning to replace the CmR cassette with AsLOV2 domain, followed by BsmBI digestion and ligation of RfxCas13d-LOV fragments to the landing pad recombination plasmid for follow-up selections.

a

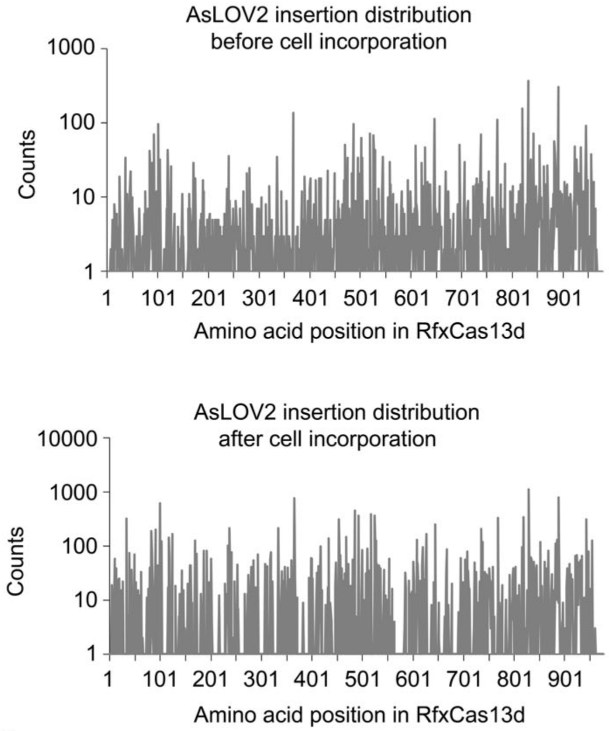

b

Insertion site	Percentages before integration	Percentages after integration
47	0.0038	0.0001
88	0.0025	0.0001
90	0.0050	0
191	0.0020	0
269	0.0020	0
391	0.0032	0
449	0.0036	0.0010
469	0.0021	0
498	0.0036	0
505	0.0059	0.0005
559	0.0052	0.0002
573	0.0013	0.0000
581	0.0007	0
582	0.0020	0
634	0.0027	0
637	0.0025	0
639	0.0025	0.0004
657	0.0023	0
674	0.0020	0
731	0.0030	0.0004
777	0.0025	0
786	0.0048	0.0005
797	0.0020	0.0002
817	0.0025	0
898	0.0018	0.0001
903	0.0027	0.0001
961	0.0027	0

c

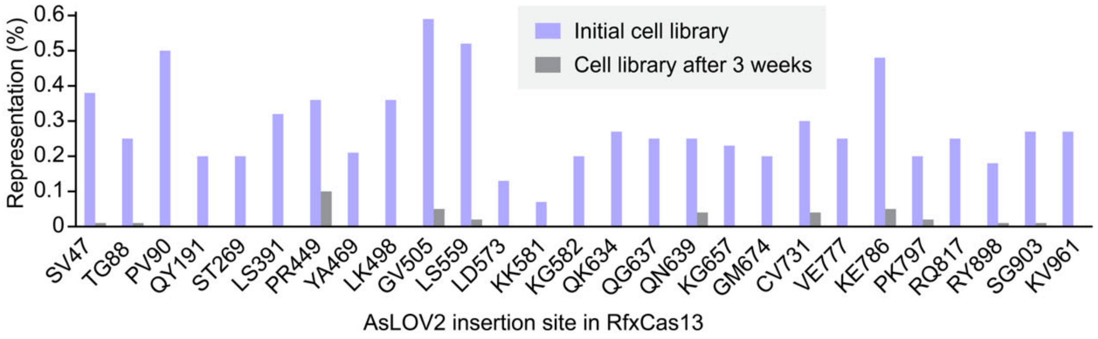

Supplementary Figure 2. Loss of some OptoCas13d library before and after cell incorporation reveals potential switchable variants. (**a**) AsLOV2 insertion distribution of RfxCas13-LOV2 library before and after cell integration as determined by next-generation sequencing. (**b**) Insertion sites disappeared after integrating the RfxCas13d-LOV2 library into the landing pad reporter cell line. (**c**) Negative selection screening identifies 27 insertion sites that might be active variants. Source data are provided as a Source Data file.

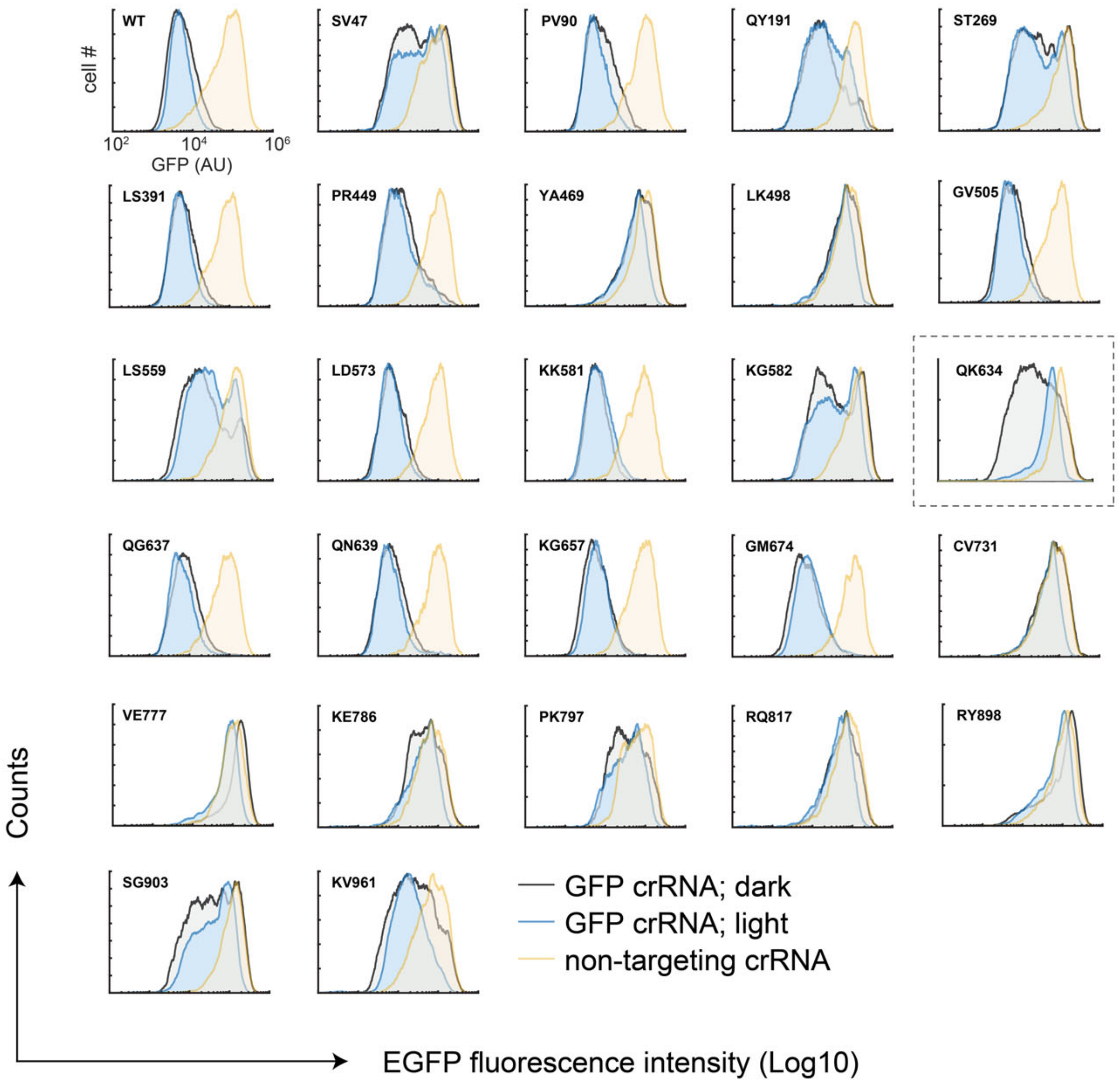

Supplementary Figure 3. Results of 26 candidates being tested. EGFP stably expressing cells were transfected with RfxCas13d with AsLOV2 insertion at different sites followed by incubation in light or dark conditions and flow cytometry analysis. Source data are provided as a Source Data file.

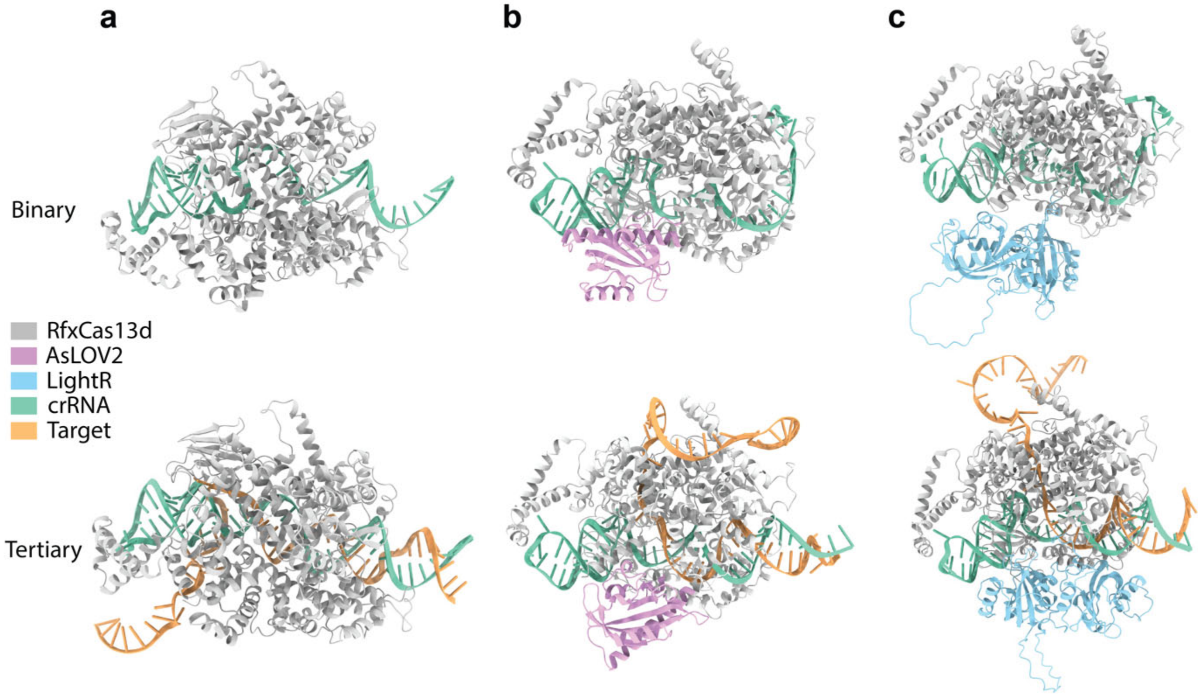

Supplementary Figure 4. **Predicted structure of binary and tertiary complexes of RfxCas13d with various inserts**. (**a**) wild-type RfxCas13d. (**b**) RfxCas13d-AsLOV2. (**c)** RfxCas13d-LightR. crRNA contains a spacer targeting EGFP mRNA. Prediction was performed using AlphaFold 3 (see **Methods**).

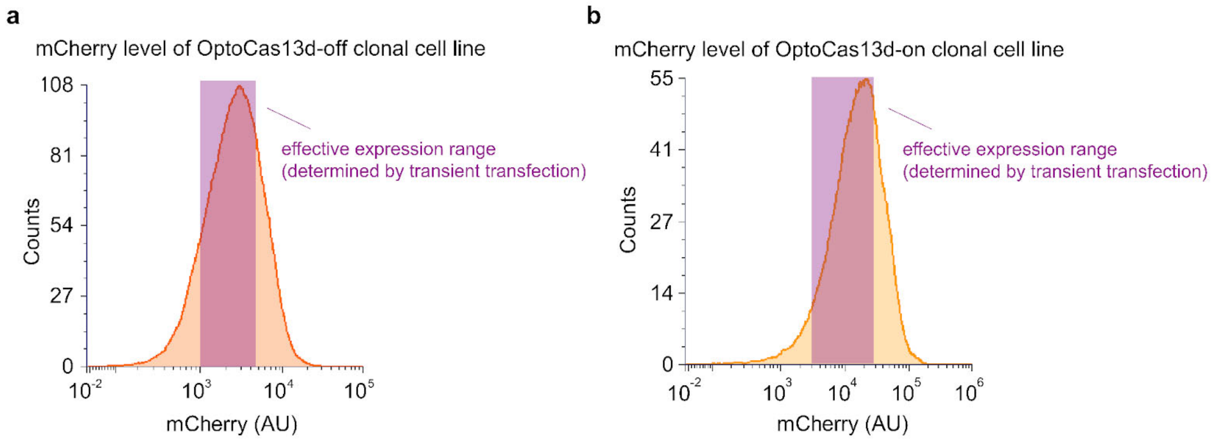

Supplementary Figure 5. Effective range of OptoCas13d-off and OptoCas13d-on expression levels that allow for robust light switchable activity. (**a**) mCherry histogram of OptoCas13d-off clonal cell line. (**b**) mCherry histogram of OptoCas13d-on clonal cell line. Both OptoCas13d-off and OptoCas13d-on were tagged with IRES-mCherry. Source data are provided as a Source Data file.

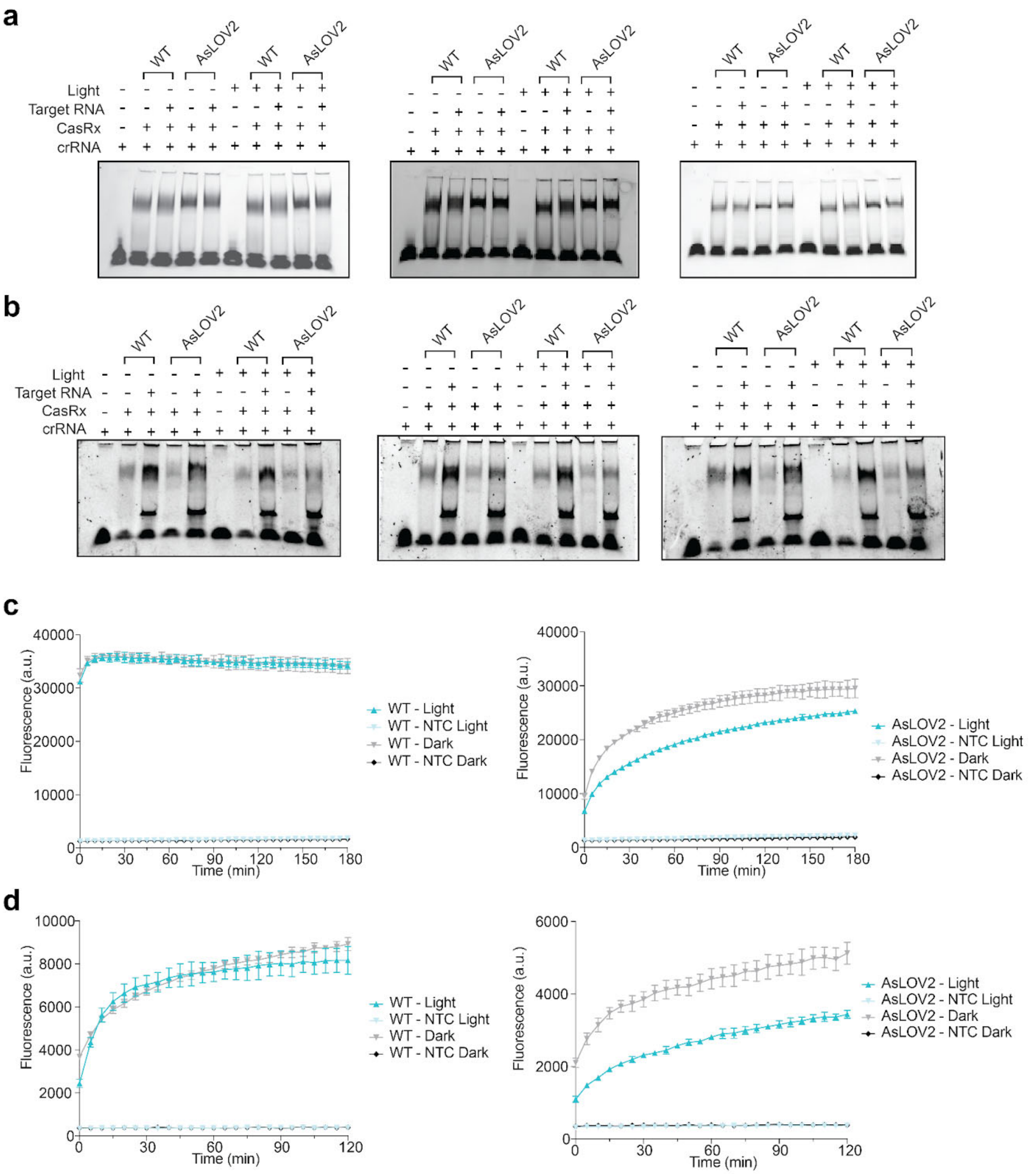

Supplementary Figure 6. *In Vitro* Characterization of Optocas13d-off and wild-type RfxCas13d. (**a**) All 3 replicates for data shown in Figure 3d. (**b**) All 3 replicates for data shown in Figure 3f. (**c**) Cleavage kinetics for WT (left) and OptoCas13d-off (right) at 126.67 nM of crRNA and 100 nM of effector. (**d**) Cleavage kinetics for WT (left) and OptoCas13d-off (right) at 6.33 nM of crRNA and 5 nM of effector. Error bars in **c-d** show mean + SD for 3 independent replicates. Source data are provided as a Source Data file.

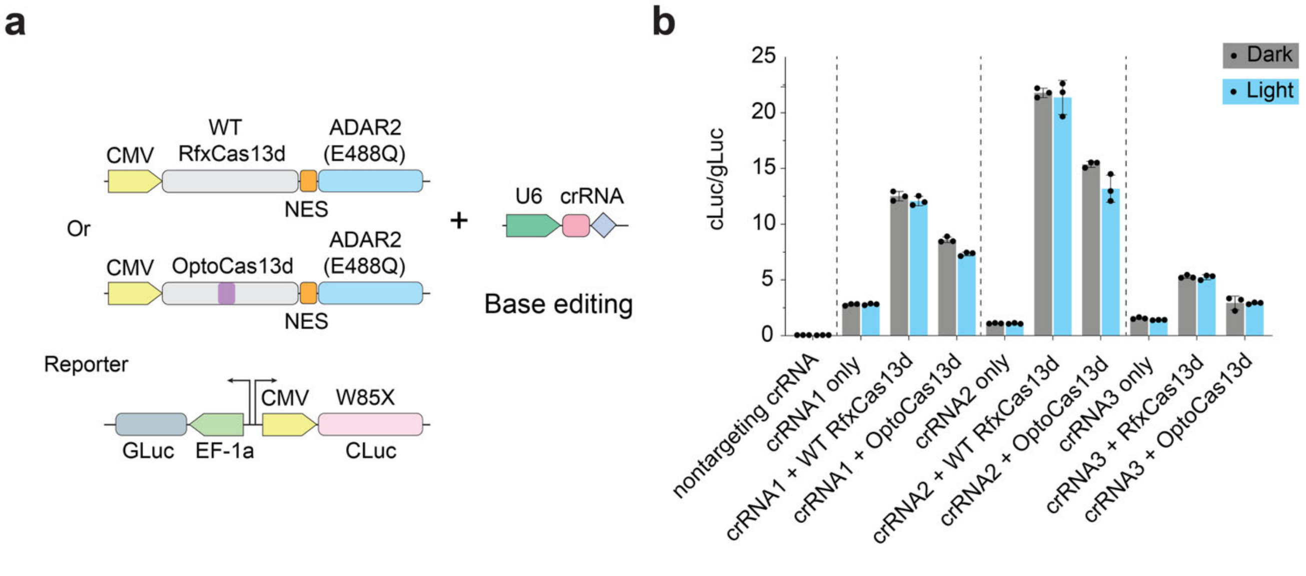

Supplementary Figure 7. A-to-I editing of deadOptoCas13d-off-ADAR fusion effector on luciferase. (**a**) Schematic of wild-type RfxCas13d and RfxCas13d-QK634-AsLOV2 constructs used for luciferase base editing. (**b**) A-to-I conversion of W85X in the CLuc mRNA for wild-type RfxCas13d and RfxCas13d-QK634-AsLOV2 in light and dark conditions. Error bars show mean + SD from n=3 independent replicates. Source data are provided as a Source Data file.

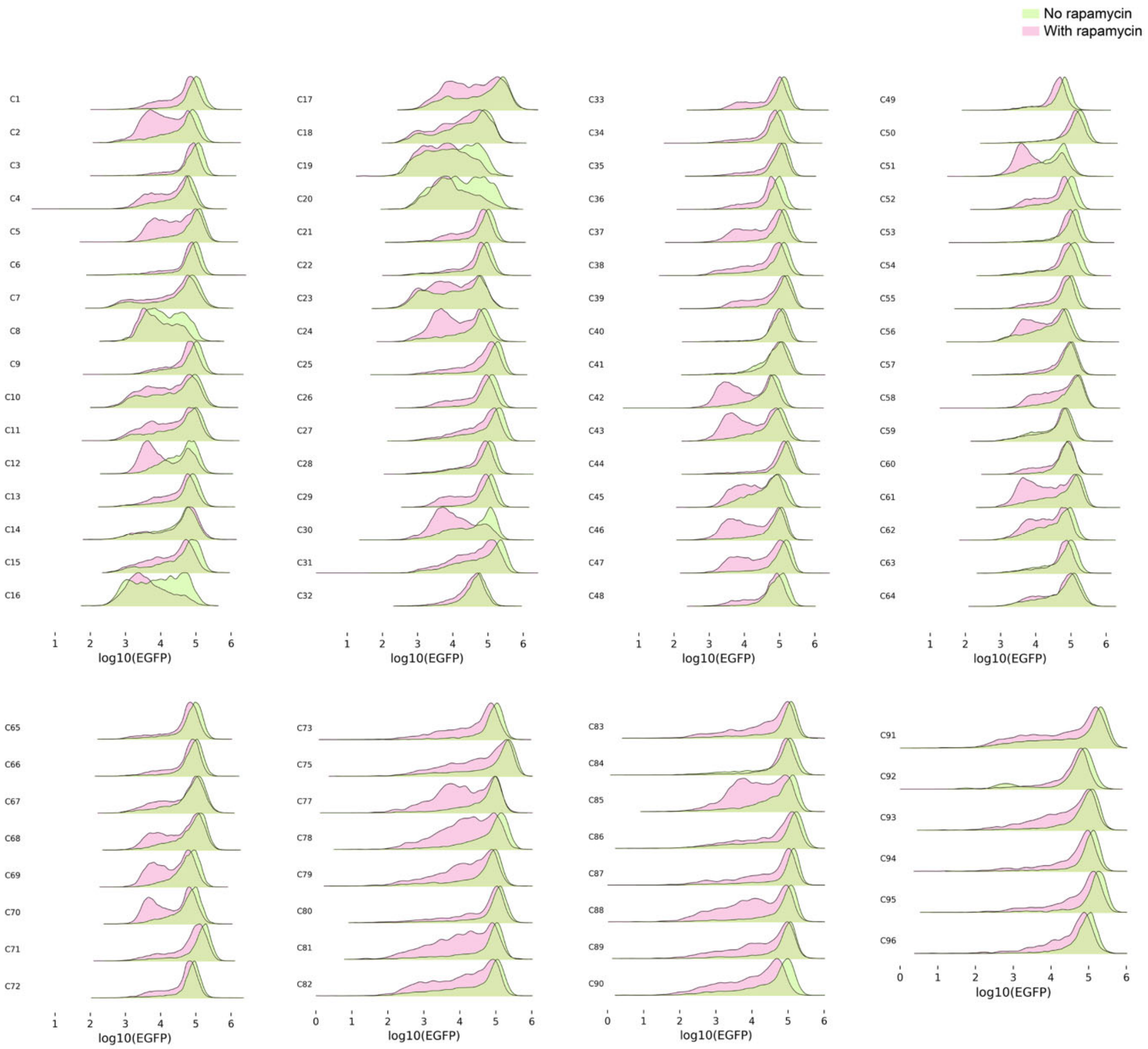

Supplementary Figure 8. Screening of 96 ChemoCas13d monoclonal cell lines for EGFP knockdown. ChemoCas13d was integrated into HEK293T with rTTA and EGFP constitutively expressed via PiggyBac™ integration. Single cells were sorted via FACS followed by transfection with crRNA targeting EGFP with/without the presence of rapamycin. Knockdown of EGFP was analyzed by flow cytometry. Clones 74 and 76 showed minimal EGFP knockdown but were excluded from above due to insufficient cells. Source data are provided as a Source Data file.

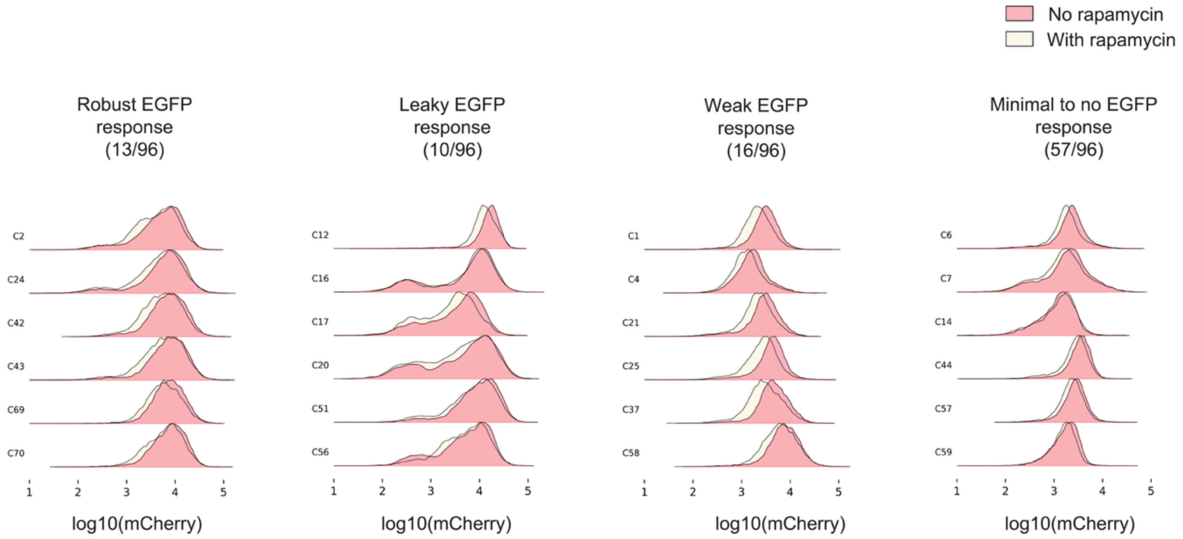

Supplementary Figure 9. Representative of single-cell mCherry expression profiles of ChemoCas13d monoclonal lines. Density plots show log10(mCherry) fluorescence, reporting doxycycline-induced ChemoCas13d expression, across monoclonal cell lines with and without rapamycin. This figure provides additional data to Fig. 5b. Source data are provided as a Source Data file.

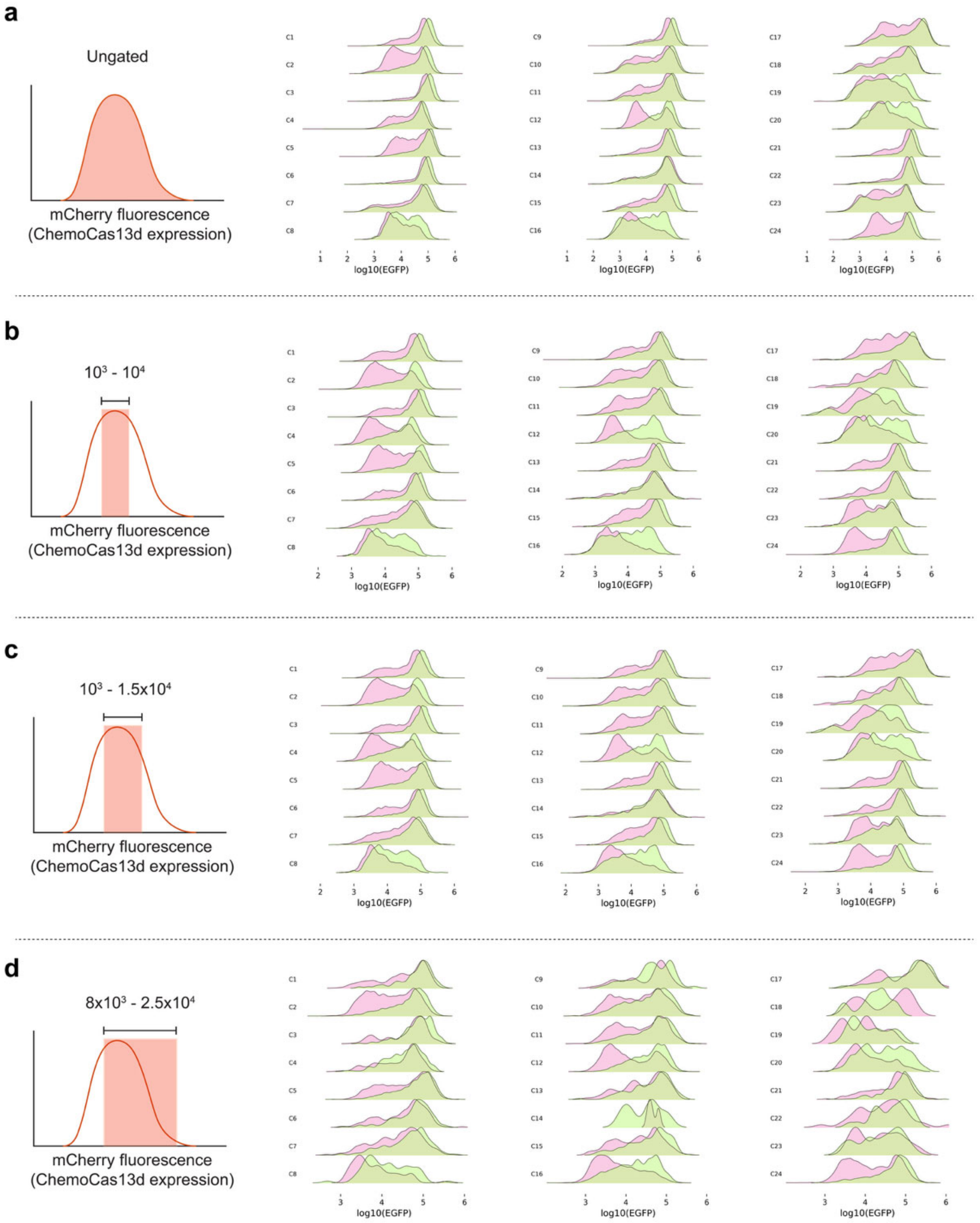

Supplementary Figure 10. Screening of 96 ChemoCas13d monoclonal cell lines for EGFP knockdown with different gating strategies for mCherry fluorescence. We sought to understand how each clone of the 96 ChemoCas13d clones (Supplementary Fig. S8) would respond to doxycycline and thereby affect EGFP knockdown even if they were derived from a single cell. Source data are provided as a Source Data file.

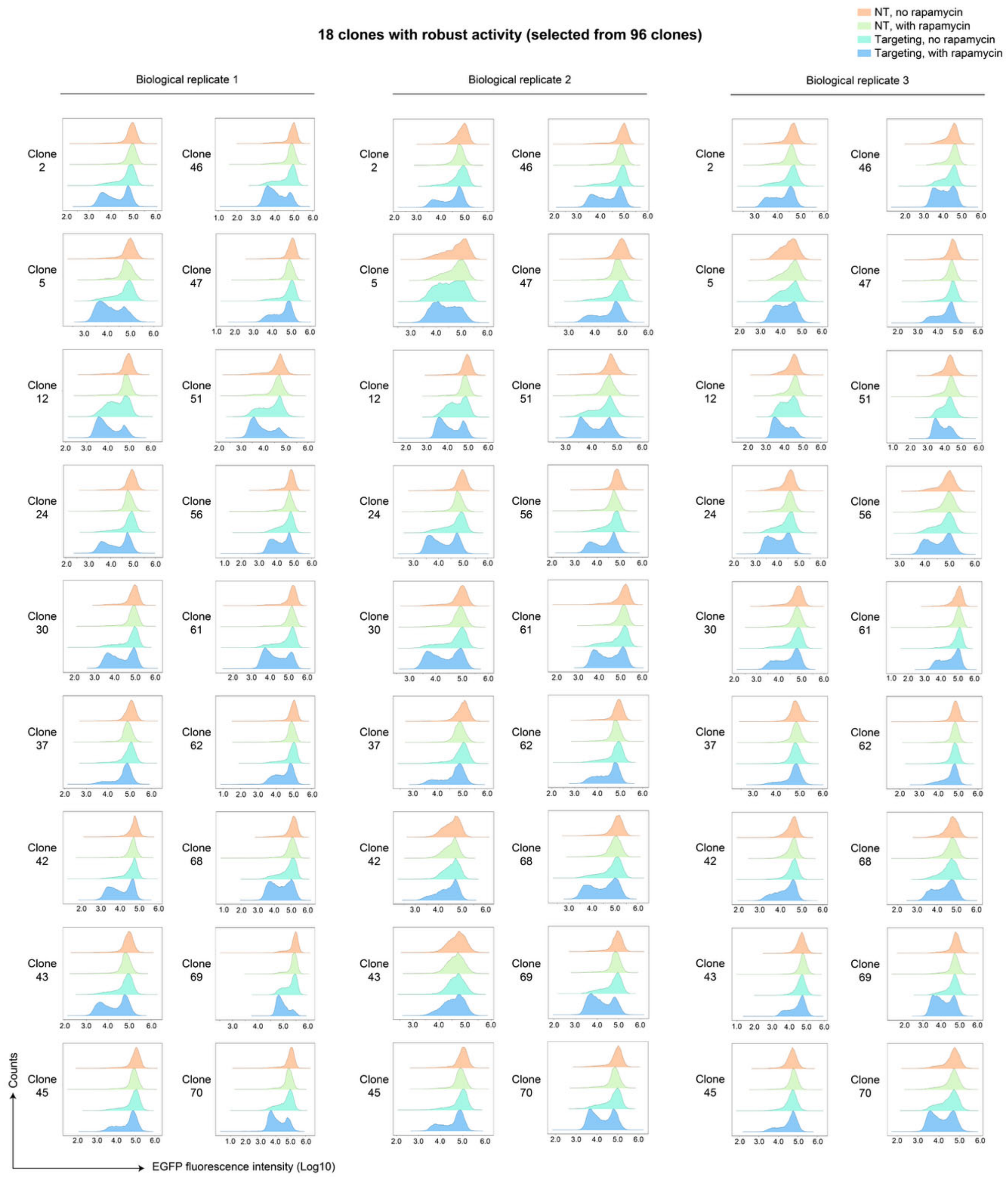

Supplementary Figure 11. Screening of 18 ChemoCas13d monoclonal cell lines showing robust EGFP knockdown. 18 ChemoCas13d monoclonal cell lines were selected from 96 clones (Figure. S8) for further characterization. The cells were transfected with crRNA targeting EGFP and non-targeting crRNA with/without the presence of rapamycin. Knockdown of EGFP was analyzed by flow cytometry. The experiments were repeated three times (n = 3 independent replicates). Source data are provided as a Source Data file.

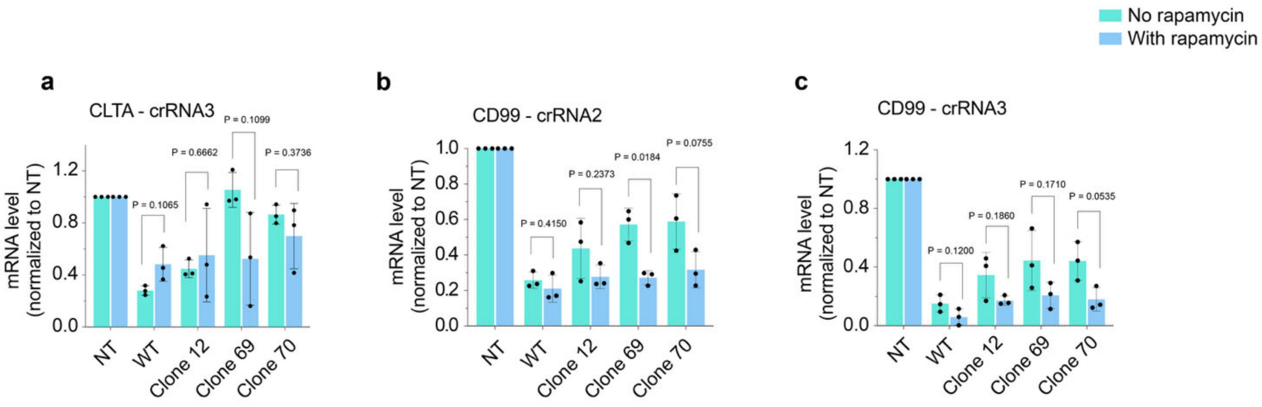

Supplementary Figure 12. Additional endogenous mRNA transcript knockdown using ChemoCas13d cell lines. (**a**)-(**c**) are supplemented to main text figure 5d-h. The experiments were repeated three times (n = 3 independent replicates). Error bars show mean + SD. Source data are provided as a Source Data file.

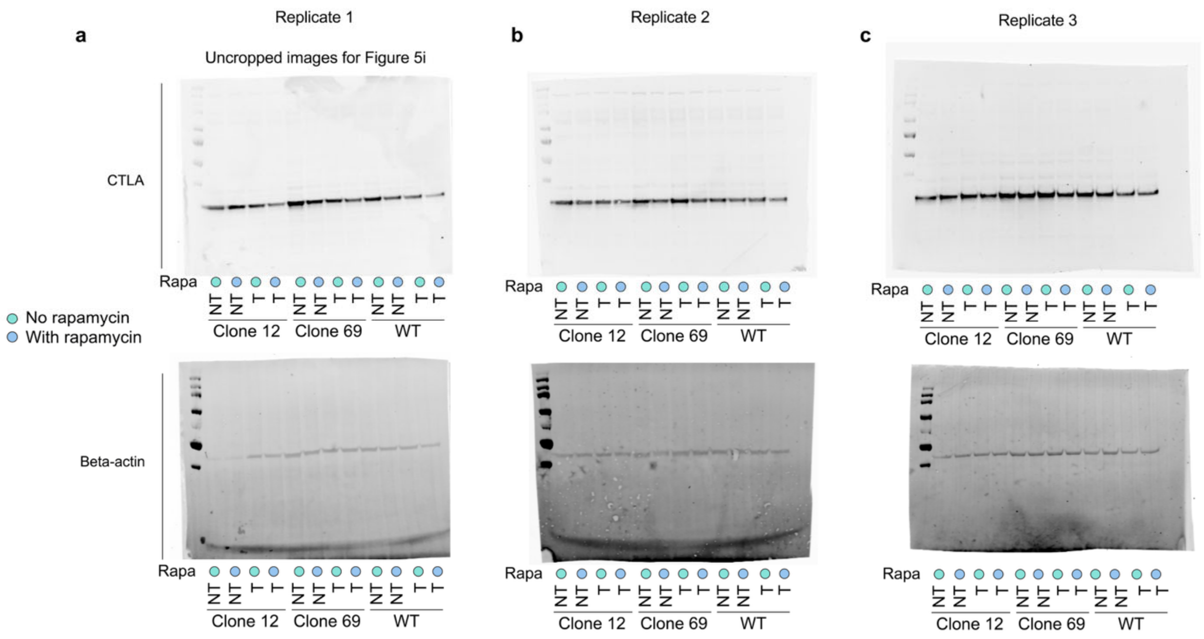

Supplementary Figure 13. Uncropped Western blots corresponding to Fig. 5h. (a)-(c) Uncropped Western blots from three independent biological replicates showing CTLA protein levels in Clone 12, Clone 69, and WT cells under non-targeting (NT) or targeting (T) conditions in the absence or presence of rapamycin (Rapa). Green circles indicate no rapamycin; blue circles indicate rapamycin treatment. CTLA signal is shown in the top panels, with β-actin serving as a loading control (bottom panels). The representative blot shown in Fig. 5i was selected from these biological replicates. Source data are provided as a Source Data file.

Supplementary Tables

Supplementary Table 1: Plasmids used for this study

Plasmids	Insert1	Insert2	Vector	Sources
pUCKanR-Mu-BsaI	Transposon-propagation vector used for Mu-BsaI modified transposon			Addgene #79769
pATT-Dest	Vector used for transposition			Addgene #79770
pXR001: EF1a-CasRx-2A-EGFP	NLS-RfxCas13d-NLS-P2A-EGFP		pXR001	Addgene #109049
pIRES2-mCherry-p53 deltaN	p53 deltaN	mCherry		Addgene #49243
LRG2.1-TagBFP2	guide RNA	TagBFP2		Addgene #124773
LightR-bRaf-mVenus	LightR-bRaf-mVenus			Addgene #162154
pUSE-Src-YF-UniRapR-mCerulean--myc	UniRapR-mCerulean			Addgene #45381
pLZD3	Non-targeting gRNA		pxr003	Addgene #109053
pLZD4	GFP-targeting gRNA		pxr003	This study
pLZD47	CasRx		PUC19	This study
pLZD78	U6_GFP-targeting gRNA	EF1a-iRFP	LRG2.1	This study
pLZD79	U6_none-targeting gRNA	EF1a-iRFP	LRG2.1	This study
pLZD81	CasRx-IRES-mCherry		AttB recombination	This study
pLZD151	CasRx-AsLOV2(QK634)-IRES-mCherry		AttB recombination	This study
pLZD182	EF1a_CasRx-IRES-mCherry		PQ	This study
pLZD183	EF1a_CasRx-AsLOV2(QK634)-IRES-mCherry		PQ	This study
pLZD185	CasRx_LightR(QK634)-IRES-mCherry		PQ	This study
pLZD189	B4GALNT1-targeting gRNA		pxr003	This study
pLZD222	AXNA4-targeting gRNA		pxr003	This study
pLZD224	AXNA4-targeting gRNA (II)		pxr003	This study

pLZD239	TREp_CasRx-LightR(QK634)-IRES-mCherry		Piggybac	This study
pLZD240	TREp_CasRx-IRES-mCherry		Piggybac	This study
pLZD241	TREp_CasRx-AsLOV2(QK634)-IRES-mCherry		Piggybac	This study
pLZD247	FTH1-targeting gRNA (II)		pxr003	This study
pLZD248	FTH1-targeting gRNA		pxr003	This study
pLZD249	CD99-targeting gRNA (II)		pxr003	This study
pLZD250	CD99-targeting gRNA		pxr003	This study
pLZD251	CD99-targeting gRNA (III)		pxr003	This study
pLZD252	CLTA-targeting gRNA (II)		pxr003	This study
pLZD253	CLTA-targeting gRNA		pxr003	This study
pLZD254	CLTA-targeting gRNA (III)		pxr003	This study
pLZD255	HECTD3-targeting gRNA		pxr003	This study
pLZD256	HECTD3-targeting gRNA (II)		pxr003	This study
pLZD257	HECTD3-targeting gRNA (III)		pxr003	This study

Supplementary Table 2: gRNA sequences

Plasmid	Description	gRNA spacer (5' to 3')
pLZD3	Non-targeting gRNA	GCTACGTGGCTTCGCCTGTCGC
pLZD4	GFP-targeting gRNA	CTTCACCTCGGCGCGGGTCTTG
pLZD189	B4GALNT1-targeting gRNA	CCTCCTGACCAGAAGCTGCCTG
pLZD222	AXNA4-targeting gRNA	CTTGTAGGCTGTCCTGATCTCC
pLZD224	AXNA4-targeting gRNA (II)	GAATGTCATCTTCAAGGCTCCG
pLZD247	FTH1-targeting gRNA (II)	GCTCTCCCAGTCATCACAGTCTGGTTTCTT
pLZD248	FTH1-targeting gRNA	CTGAAGGAAGATTCGGCCACCTCGTTGGTT
pLZD249	CD99-targeting gRNA (II)	AACGCCATCCGCAAGGTCAGCATCTGAAAA
pLZD250	CD99-targeting gRNA	GTCAGCATCTGAAAAGCTACCGGAGGAACT
pLZD251	CD99-targeting gRNA (III)	ATGGCTCCAGCCACGGCGACCACGACAGCC
pLZD252	CLTA-targeting gRNA (II)	GGAGATGAGGACTGAGCGCATGCGGGAGAC
pLZD253	CLTA-targeting gRNA	TCCACTCTGCTTCTTGCTTCCGAGAATTGG
pLZD254	CLTA-targeting gRNA (III)	GTGGCTCTTCAGTGCACCAGCGGGGCCTGC
pLZD255	HECTD3-targeting gRNA	TGGTATCCACTGTGAGTAGCAGCTTCTTGA
pLZD256	HECTD3-targeting gRNA (II)	GCTCTCCACATACTGCTTCACGCTACCCAG
pLZD257	HECTD3-targeting gRNA (III)	GCGTCTGTGGTCTCGTAGCCCAGCTTGTCT

Supplementary Table 3: qPCR primers

Transcripts	Forward primer (5'-3')	Reverse primer (5'-3')
GAPDH	ACAACTTTGGTATCGTGGAAGG	GCCATCACGCCACAGTTTC
GFP	AAGCAGAAGAACGGCATCAA	GGGGGTGTTCTGCTGGTAGT
B4GALNT1	TGAGGCTGCTTTCACTATCCGC	GAGGAAGGTCTTGGTGGCAATC
AXNA4	GATGCTCTCGTGAGACAGGATG	GCAACAGGTGATTTCGGTTCCG
FTH1	TGAAGCTGCAGAACCAACGAGG	GCACACTCCATTGCATTCAGCC
CD99	GAAAAGGAGGCAGTGATGGTGG	TGAAGCTAGAGATGGCTCCAGC
CLTA	CGATTGCAGTCAGAGCCTGAAAG	TAGCTGCTCGTCCTGTCTTGCA
HECTD3	ATCGAGATCCGCATCGTGGAGT	TAGTTGGCTGGAACAGGTCTGC

Supplementary Notes

Supplementary Note 1: sequence of RfxCas13d-AsLOV2(QK634)

In black: RfxCas13d   In green: Linker   In blue: AsLOV2 (408-543)

ATCGAAAAAAAAAAGTCCTTCGCCAAGGGCATGGGCGTGAAGTCCACACTCGTGTCCGGCTCCAAAGTGTACATGACAACCTTCG
CCGAAGGCAGCGACGCCAGGCTGGAAAAGATCGTGGAGGGCGACAGCATCAGGAGCGTGAATGAGGGCGAGGCCTTCAGCGCTGA
AATGGCCGATAAAAACGCCGGCTATAAGATCGGCAACGCCAAATTCAGCCATCCTAAGGGCTACGCCGTGGTGGCTAACAACCCT
CTGTATACAGGACCCGTCCAGCAGGATATGCTCGGCCTGAAGGAAACTCTGGAAAAGAGGTACTTCGGCGAGAGCGCTGATGGCA
ATGACAATATTTGTATCCAGGTGATCCATAACATCCTGGACATTGAAAAAATCCTCGCCGAATACATTACCAACGCCGCCTACGC
CGTCAACAATATCTCCGGCCTGGATAAGGACATTATTGGATTCGGCAAGTTCTCCACAGTGTATACCTACGACGAATTCAAAGAC
CCCGAGCACCATAGGGCCGCTTTCAACAATAACGATAAGCTCATCAACGCCATCAAGGCCCAGTATGACGAGTTCGACAACTTCC
TCGATAACCCCAGACTCGGCTATTTCGGCCAGGCCTTTTTCAGCAAGGAGGGCAGAAATTACATCATCAATTACGGCAACGAATG
CTATGACATTCTGGCCCTCCTGAGCGGACTGAGGCACTGGGTGGTCCATAACAACGAAGAAGAGTCCAGGATCTCCAGGACCTGG
CTCTACAACCTCGATAAGAACCTCGACAACGAATACATCTCCACCCTCAACTACCTCTACGACAGGATCACCAATGAGCTGACCA
ACTCCTTCTCCAAGAACTCCGCCGCCAACGTGAACTATATTGCCGAAACTCTGGGAATCAACCCTGCCGAATTCGCCGAACAATA
TTTCAGATTCAGCATTATGAAAGAGCAGAAAAACCTCGGATTCAATATCACCAAGCTCAGGGAAGTGATGCTGGACAGGAAGGAT
ATGTCCGAGATCAGGAAAAATCATAAGGTGTTCGACTCCATCAGGACCAAGGTCTACACCATGATGGACTTTGTGATTTATAGGT
ATTACATCGAAGAGGATGCCAAGGTGGCTGCCGCCAATAAGTCCCTCCCCGATAATGAGAAGTCCCTGAGCGAGAAGGATATCTT
TGTGATTAACCTGAGGGGCTCCTTCAACGACGACCAGAAGGATGCCCTCTACTACGATGAAGCTAATAGAATTTGGAGAAAGCTC
GAAAATATCATGCACAACATCAAGGAATTTAGGGGAAACAAGACAAGAGAGTATAAGAAGAAGGACGCCCCTAGACTGCCCAGAA
TCCTGCCCGCTGGCCGTGATGTTTCCGCCTTCAGCAAACTCATGTATGCCCTGACCATGTTCCTGGATGGCAAGGAGATCAACGA
CCTCCTGACCACCCTGATTAATAAATTCGATAACATCCAGAGCTTCCTGAAGGTGATGCCTCTCATCGGAGTCAACGCTAAGTTC
GTGGAGGAATACGCCTTTTTCAAAGACTCCGCCAAGATCGCCGATGAGCTGAGGCTGATCAAGTCCTTCGCTAGAATGGGAGAAC
CTATTGCCGATGCCAGGAGGGCCATGTATATCGACGCCATCCGTATTTTAGGAACCAACCTGTCCTATGATGAGCTCAAGGCCCT
CGCCGACACCTTTTCCCTGGACGAGAACGGAAACAAGCTCAAGAAAGGCAAGCACGGCATGAGAAATTTCATTATTAATAACGTG
ATCAGCAATAAAAGGTTCCACTACCTGATCAGATACGGTGATCCTGCCCACCTCCATGAGATCGCCAAAAACGAGGCCGTGGTGA
AGTTCGTGCTCGGCAGGATCGCTGACATCCAGATGCATCTTTTGGAACGTATCGAAAAGAATTTTGTCATCACGGATCCGCGTCT
TCCCGACAATCCGATTATCTTCGCGTCAGACTCTTTCTTACAACTGACTGAGTATAGTAGAGAGGAGATATTGGGGCGTAACTGT
AGATTTCTTCAGGGGCCAGAAACTGATCGGGCTACCGTTCGCAAGATACGTGACGCAATAGACAACCAGACCGAGGTGACGGTGC
AGCTGATTAACTACACAAAGTCTGGGAAGAAGTTCTGGAACCTGTTTCATTTACAACCTATGAGAGATCAAAAAGGTGACGTTCA
ATATTTCATCGGGGTTCAGTTAGATGGGACTGAGCACGTGAGAGATGCAGCAGAAAGAGAGGGTGTAATGCTTATTAAAAAAACA
GCCGAGAATATCGACGAAGCCGCTAAGCGTCACCAGAAAAAACAGGGCCAGAACGGCAAGAACCAGATCGACAGGTACTACGAAA
CTTGTATCGGAAAGGATAAGGGCAAGAGCGTGAGCGAAAAGGTGGACGCTCTCACAAAGATCATCACCGGAATGAACTACGACCA
ATTCGACAAGAAAAGGAGCGTCATTGAGGACACCGGCAGGGAAAACGCCGAGAGGGAGAAGTTTAAAAAGATCATCAGCCTGTAC
CTCACCGTGATCTACCACATCCTCAAGAATATTGTCAATATCAACGCCAGGTACGTCATCGGATTCCATTGCGTCGAGCGTGATG
CTCAACTGTACAAGGAGAAAGGCTACGACATCAATCTCAAGAAACTGGAAGAGAAGGGATTCAGCTCCGTCACCAAGCTCTGCGC
TGGCATTGATGAAACTGCCCCCGATAAGAGAAAGGACGTGGAAAAGGAGATGGCTGAAAGAGCCAAGGAGAGCATTGACAGCCTC
GAGAGCGCCAACCCCAAGCTGTATGCCAATTACATCAAATACAGCGACGAGAAGAAAGCCGAGGAGTTCACCAGGCAGATTAACA
GGGAGAAGGCCAAAACCGCCCTGAACGCCTACCTGAGGAACACCAAGTGGAATGTGATCATCAGGGAGGACCTCCTGAGAATTGA
CAACAAGACATGTACCCTGTTCAGAAACAAGGCCGTCCACCTGGAAGTGGCCAGGTATGTCCACGCCTATATCAACGACATTGCC
GAGGTCAATTCCTACTTCCAACTGTACCATTACATCATGCAGAGAATTATCATGAATGAGAGGTACGAGAAAAGCAGCGGAAAGG
TGTCCGAGTACTTCGACGCTGTGAATGACGAGAAGAAGTACAACGATAGGCTCCTGAAACTGCTGTGTGTGCCTTTCGGCTACTG
TATCCCCAGGTTTAAGAACCTGAGCATCGAGGCCCTGTTCGATAGGAACGAGGCCGCCAAGTTCGACAAGGAGAAAAAGAAGGTG
TCCGGCAATTCC

Supplementary Note 2: sequence of RfxCas13d-LightR(QK634)

In black: RfxCas13d   In blue: LightR

ATCGAAAAAAAAAAGTCCTTCGCCAAGGGCATGGGCGTGAAGTCCACACTCGTGTCCGGCTCCAAAGTGTACATGACAACCTTCG
CCGAAGGCAGCGACGCCAGGCTGGAAAAGATCGTGGAGGGCGACAGCATCAGGAGCGTGAATGAGGGCGAGGCCTTCAGCGCTGA
AATGGCCGATAAAAACGCCGGCTATAAGATCGGCAACGCCAAATTCAGCCATCCTAAGGGCTACGCCGTGGTGGCTAACAACCCT
CTGTATACAGGACCCGTCCAGCAGGATATGCTCGGCCTGAAGGAAACTCTGGAAAAGAGGTACTTCGGCGAGAGCGCTGATGGCA
ATGACAATATTTGTATCCAGGTGATCCATAACATCCTGGACATTGAAAAAATCCTCGCCGAATACATTACCAACGCCGCCTACGC
CGTCAACAATATCTCCGGCCTGGATAAGGACATTATTGGATTCGGCAAGTTCTCCACAGTGTATACCTACGACGAATTCAAAGAC
CCCGAGCACCATAGGGCCGCTTTCAACAATAACGATAAGCTCATCAACGCCATCAAGGCCCAGTATGACGAGTTCGACAACTTCC
TCGATAACCCCAGACTCGGCTATTTCGGCCAGGCCTTTTTCAGCAAGGAGGGCAGAAATTACATCATCAATTACGGCAACGAATG
CTATGACATTCTGGCCCTCCTGAGCGGACTGAGGCACTGGGTGGTCCATAACAACGAAGAAGAGTCCAGGATCTCCAGGACCTGG
CTCTACAACCTCGATAAGAACCTCGACAACGAATACATCTCCACCCTCAACTACCTCTACGACAGGATCACCAATGAGCTGACCA
ACTCCTTCTCCAAGAACTCCGCCGCCAACGTGAACTATATTGCCGAAACTCTGGGAATCAACCCTGCCGAATTCGCCGAACAATA
TTTCAGATTCAGCATTATGAAAGAGCAGAAAAACCTCGGATTCAATATCACCAAGCTCAGGGAAGTGATGCTGGACAGGAAGGAT
ATGTCCGAGATCAGGAAAAATCATAAGGTGTTCGACTCCATCAGGACCAAGGTCTACACCATGATGGACTTTGTGATTTATAGGT
ATTACATCGAAGAGGATGCCAAGGTGGCTGCCGCCAATAAGTCCCTCCCCGATAATGAGAAGTCCCTGAGCGAGAAGGATATCTT
TGTGATTAACCTGAGGGGCTCCTTCAACGACGACCAGAAGGATGCCCTCTACTACGATGAAGCTAATAGAATTTGGAGAAAGCTC
GAAAATATCATGCACAACATCAAGGAATTTAGGGGAAACAAGACAAGAGAGTATAAGAAGAAGGACGCCCCTAGACTGCCCAGAA
TCCTGCCCGCTGGCCGTGATGTTTCCGCCTTCAGCAAACTCATGTATGCCCTGACCATGTTCCTGGATGGCAAGGAGATCAACGA
CCTCCTGACCACCCTGATTAATAAATTCGATAACATCCAGAGCTTCCTGAAGGTGATGCCTCTCATCGGAGTCAACGCTAAGTTC
GTGGAGGAATACGCCTTTTTCAAAGACTCCGCCAAGATCGCCGATGAGCTGAGGCTGATCAAGTCCTTCGCTAGAATGGGAGAAC
CTATTGCCGATGCCAGGAGGGCCATGTATATCGACGCCATCCGTATTTTAGGAACCAACCTGTCCTATGATGAGCTCAAGGCCCT
CGCCGACACCTTTTCCCTGGACGAGAACGGAAACAAGCTCAAGAAAGGCAAGCACGGCATGAGAAATTTCATTATTAATAACGTG
ATCAGCAATAAAAGGTTCCACTACCTGATCAGATACGGTGATCCTGCCCACCTCCATGAGATCGCCAAAAACGAGGCCGTGGTGA
AGTTCGTGCTCGGCAGGATCGCTGACATCCAGggaccaggtggcagcggaggtcataccttgtatgcgccggggggttatgacat
catgggttacctcatacagatcatgaataggccgaacccacaagtggagctcggacccgtcgatacctcctgcgctctcattctg
tgtgaccttaagcagaaggataccccctatcgtgtacgcctccgaggcatttctgtacatgacagggtactcgaacgccgaagtgc
tgggacggaactgccgcttcctgcaaagcccggatggaatggtgaagcctaagtcaacccgcaaatacgtggactccaacactat
caacaccatgcgcaaggccattgaccgcaatgctgaggtgcaagtggaagtggtgaacttcaagaagaatggacagcgcttcgtc
aacttcctgactatgatccccgtgcgcgacgagaccggcgaataccggtacagcatggggtttcagtgtgagacagagggcggct
ccggaggcagcggcggttctggaggttccggtggcggctccggaggtagcggagggtctcacactctttacgcccctggaggata
cgacattatgggatatttgattcagattatgaaccgcccaaaccctcaggtcgaactggggcctgtggacacgtcatgtgccctg
atcctgtgcgatctgaagcaaaaggacactccgattgtctacgcctcggaagccttcttgtatatgaccggatacagcaatgcag
aggtgctcggtaggaactgcagattcctgcagtcccccgacgggatggtgaaaccaaagtcgactcgcaaatatgtggactcgaa
cacgatcaatacaatgcggaaggccatcgaccggaacgccgaggtccaggtggaggtggtcaactttaagaagaacggccagcgg
ttcgtgaactttctcaccatgattccggtccgggatgaaaccggagagtacagatactccatgggattccagtgcgaaaccgaag
ggtccggaggtcccggaAAAAAACAGGGCCAGAACGGCAAGAACCAGATCGACAGGTACTACGAAACTTGTATCGGAAAGGATAA
GGGCAAGAGCGTGAGCGAAAAGGTGGACGCTCTCACAAAGATCATCACCGGAATGAACTACGACCAATTCGACAAGAAAAGGAGC
GTCATTGAGGACACCGGCAGGGAAAACGCCGAGAGGGAGAAGTTTAAAAAGATCATCAGCCTGTACCTCACCGTGATCTACCACA
TCCTCAAGAATATTGTCAATATCAACGCCAGGTACGTCATCGGATTCCATTGCGTCGAGCGTGATGCTCAACTGTACAAGGAGAA
AGGCTACGACATCAATCTCAAGAAACTGGAAGAGAAGGGATTCAGCTCCGTCACCAAGCTCTGCGCTGGCATTGATGAAACTGCC
CCCGATAAGAGAAAGGACGTGGAAAAGGAGATGGCTGAAAGAGCCAAGGAGAGCATTGACAGCCTCGAGAGCGCCAACCCCAAGC
TGTATGCCAATTACATCAAATACAGCGACGAGAAGAAAGCCGAGGAGTTCACCAGGCAGATTAACAGGGAGAAGGCCAAAACCGC
CCTGAACGCCTACCTGAGGAACACCAAGTGGAATGTGATCATCAGGGAGGACCTCCTGAGAATTGACAACAAGACATGTACCCTG
TTCAGAAACAAGGCCGTCCACCTGGAAGTGGCCAGGTATGTCCACGCCTATATCAACGACATTGCCGAGGTCAATTCCTACTTCC
AACTGTACCATTACATCATGCAGAGAATTATCATGAATGAGAGGTACGAGAAAAGCAGCGGAAAGGTGTCCGAGTACTTCGACGC
TGTGAATGACGAGAAGAAGTACAACGATAGGCTCCTGAAACTGCTGTGTGTGCCTTTCGGCTACTGTATCCCCAGGTTTAAGAAC
CTGAGCATCGAGGCCCTGTTCGATAGGAACGAGGCCGCCAAGTTCGACAAGGAGAAAAAGAAGGTGTCCGGCAATTCC

Supplementary Note 3: sequence of RfxCas13d-UniRapR (QK634)

In black: RfxCas13d   In brown: UniRapR

ATCGAAAAAAAAAAGTCCTTCGCCAAGGGCATGGGCGTGAAGTCCACACTCGTGTCCGGCTCCAAAGTGTACATGACAACCTTCG
CCGAAGGCAGCGACGCCAGGCTGGAAAAGATCGTGGAGGGCGACAGCATCAGGAGCGTGAATGAGGGCGAGGCCTTCAGCGCTGA
AATGGCCGATAAAAACGCCGGCTATAAGATCGGCAACGCCAAATTCAGCCATCCTAAGGGCTACGCCGTGGTGGCTAACAACCCT
CTGTATACAGGACCCGTCCAGCAGGATATGCTCGGCCTGAAGGAAACTCTGGAAAAGAGGTACTTCGGCGAGAGCGCTGATGGCA
ATGACAATATTTGTATCCAGGTGATCCATAACATCCTGGACATTGAAAAAATCCTCGCCGAATACATTACCAACGCCGCCTACGC
CGTCAACAATATCTCCGGCCTGGATAAGGACATTATTGGATTCGGCAAGTTCTCCACAGTGTATACCTACGACGAATTCAAAGAC
CCCGAGCACCATAGGGCCGCTTTCAACAATAACGATAAGCTCATCAACGCCATCAAGGCCCAGTATGACGAGTTCGACAACTTCC
TCGATAACCCCAGACTCGGCTATTTCGGCCAGGCCTTTTTCAGCAAGGAGGGCAGAAATTACATCATCAATTACGGCAACGAATG
CTATGACATTCTGGCCCTCCTGAGCGGACTGAGGCACTGGGTGGTCCATAACAACGAAGAAGAGTCCAGGATCTCCAGGACCTGG
CTCTACAACCTCGATAAGAACCTCGACAACGAATACATCTCCACCCTCAACTACCTCTACGACAGGATCACCAATGAGCTGACCA
ACTCCTTCTCCAAGAACTCCGCCGCCAACGTGAACTATATTGCCGAAACTCTGGGAATCAACCCTGCCGAATTCGCCGAACAATA
TTTCAGATTCAGCATTATGAAAGAGCAGAAAAACCTCGGATTCAATATCACCAAGCTCAGGGAAGTGATGCTGGACAGGAAGGAT
ATGTCCGAGATCAGGAAAAATCATAAGGTGTTCGACTCCATCAGGACCAAGGTCTACACCATGATGGACTTTGTGATTTATAGGT
ATTACATCGAAGAGGATGCCAAGGTGGCTGCCGCCAATAAGTCCCTCCCCGATAATGAGAAGTCCCTGAGCGAGAAGGATATCTT
TGTGATTAACCTGAGGGGCTCCTTCAACGACGACCAGAAGGATGCCCTCTACTACGATGAAGCTAATAGAATTTGGAGAAAGCTC
GAAAATATCATGCACAACATCAAGGAATTTAGGGGAAACAAGACAAGAGAGTATAAGAAGAAGGACGCCCCTAGACTGCCCAGAA
TCCTGCCCGCTGGCCGTGATGTTTCCGCCTTCAGCAAACTCATGTATGCCCTGACCATGTTCCTGGATGGCAAGGAGATCAACGA
CCTCCTGACCACCCTGATTAATAAATTCGATAACATCCAGAGCTTCCTGAAGGTGATGCCTCTCATCGGAGTCAACGCTAAGTTC
GTGGAGGAATACGCCTTTTTCAAAGACTCCGCCAAGATCGCCGATGAGCTGAGGCTGATCAAGTCCTTCGCTAGAATGGGAGAAC
CTATTGCCGATGCCAGGAGGGCCATGTATATCGACGCCATCCGTATTTTAGGAACCAACCTGTCCTATGATGAGCTCAAGGCCCT
CGCCGACACCTTTTCCCTGGACGAGAACGGAAACAAGCTCAAGAAAGGCAAGCACGGCATGAGAAATTTCATTATTAATAACGTG
ATCAGCAATAAAAGGTTCCACTACCTGATCAGATACGGTGATCCTGCCCACCTCCATGAGATCGCCAAAAACGAGGCCGTGGTGA
AGTTCGTGCTCGGCAGGATCGCTGACATCCAGggaccaggtacctgcgtggtgcactacaccgggatgcttgaagatggaaagaa
atttgattcctcccgggacagaaacaagccctttaagtttatgctaggcaagcaggaggtgatccgaggctgggaagaaggggtt
gcccagatgagtgtgggtcagagagccaaactgactatatctccagattatgcctatggtgccactgggcacggttcgggctccg
gatcaggcgtcaaggacctcctccaagcctgggacctctattatcatgtgttccgacgaatctcaggtcctccaggacctggatc
aggtctctggcatgagatgtggcatgaaggcctggaagaggcatctcgtttgtactttggggaaaggaacgtgaaaggcatgttt
gaggtgctggagcccttgcatgctatgatggaacggggccccagactctgaaggaaacatcctttaatcaggcctatggtcgag
atttaatggaggcccaagagtggtgcaggaagtacatgaaatcagggtcatcaggggggctccggatcaggcatcatcccaccaca
tgccactctcgtcttcgatgtggagcttctaaaactggaaggtcccggaAAAAAACAGGGCCAGAACGGCAAGAACCAGATCGAC
AGGTACTACGAAACTTGTATCGGAAAGGATAAGGGCAAGAGCGTGAGCGAAAAGGTGGACGCTCTCACAAAGATCATCACCGGAA
TGAACTACGACCAATTCGACAAGAAAAGGAGCGTCATTGAGGACACCGGCAGGGAAAACGCCGAGAGGGAGAAGTTTAAAAAGAT
CATCAGCCTGTACCTCACCGTGATCTACCACATCCTCAAGAATATTGTCAATATCAACGCCAGGTACGTCATCGGATTCCATTGC
GTCGAGCGTGATGCTCAACTGTACAAGGAGAAAGGCTACGACATCAATCTCAAGAAACTGGAAGAGAAGGGATTCAGCTCCGTCA
CCAAGCTCTGCGCTGGCATTGATGAAACTGCCCCCGATAAGAGAAAGGACGTGGAAAAGGAGATGGCTGAAAGAGCCAAGGAGAG
CATTGACAGCCTCGAGAGCGCCAACCCCAAGCTGTATGCCAATTACATCAAATACAGCGACGAGAAGAAAGCCGAGGAGTTCACC
AGGCAGATTAACAGGGAGAAGGCCAAAACCGCCCTGAACGCCTACCTGAGGAACACCAAGTGGAATGTGATCATCAGGGAGGACC
TCCTGAGAATTGACAACAAGACATGTACCCTGTTCAGAAACAAGGCCGTCCACCTGGAAGTGGCCAGGTATGTCCACGCCTATAT
CAACGACATTGCCGAGGTCAATTCCTACTTCCAACTGTACCATTACATCATGCAGAGAATTATCATGAATGAGAGGTACGAGAAA
AGCAGCGGAAAGGTGTCCGAGTACTTCGACGCTGTGAATGACGAGAAGAAGTACAACGATAGGCTCCTGAAACTGCTGTGTGTGC
CTTTCGGCTACTGTATCCCCAGGTTTAAGAACCTGAGCATCGAGGCCCTGTTCGATAGGAACGAGGCCGCCAAGTTCGACAAGGA
GAAAAAGAAGGTGTCCGGCAATTCC
