# Supplementary material for: Multimodal control of Cas13d activity through domain insertion at an allosteric hotspot
Source: Nat Commun. 2026 Jun 3;17:7146. doi: 10.1038/s41467-026-73645-5 (PMC13396516; doi:10.1038/s41467-026-73645-5)
Supplement: Supplementary file 3 — Transparent Peer Review file [file 41467_2026_73645_MOESM3_ESM.pdf]

nature portfolio

Peer Review File

Multimodal control of Cas13 activity through domain insertion at an allosteric hotspot

Corresponding Author: Dr Jared Toettcher

This file contains all reviewer reports in order by version, followed by all author rebuttals in order by version.

Version 0:

Reviewer comments:

Reviewer #1

(Remarks to the Author)
This manuscript describes construction of an optogenetic Cas13 (RfxCas13d), a RNA-targeting CRISPR effector protein, using AsLOV2 insertion. The authors use a screening strategy to identify candidate insertion sites, then test activity of candidates in light and dark for ability to block constitutive EGFP expression to identify a top candidate that shows light-dark differences in function. The authors attempt to characterize mechanistically how the AsLOV2 insertion impacts CRISPR activity, observing minimal effect on complex assembly, implying an effect on nuclease activity, and swap out the AsLOV2 for other regulatory domain insertions. There are several things done well in this manuscript: it is well-written, incorporates an interesting screen technology, uses rigorous approaches, and the data is convincing. The mechanistic studies add to an understanding of how the insertion site affects activity, and the authors demonstrate the ability to regulate expression at endogenous loci. While the study seems appropriate for a more specialized journal as is, it has several weaknesses that make it seem premature for publication in a moderately high impact journal such as Nat. Comm. The main limitation is the engineered protein shows only modest light regulation. The authors overcome this limitation by sorting to select cells that optimally express the effector protein at an intermediate level, prior to experimentally testing for light-dark differences, however the requirement for sorting impedes practical use. To improve impact, the authors could incorporate optimization steps (for example, tinkering with the linkers flanking the inserted domains) to try to improve outcomes. As another way to improve impact, applying the system in a model organism could mitigate concerns that the degree of light regulation is insufficient for practical use.

Additional points: Line 227. More explanation of the trans-cleavage assay would be helpful, for example describing how fluorescence is related to activity in this assay. Also, which reference is relevant to this assay? The text refers to ref 29 , while the methods refer to ref 27.
Panel 2c, Please indicate the value at the origin of the Y-axis.
The manuscript mentions a cell line expressing EGFP but on line 328 notes use of EGFP-PEST. Is EGFP-PEST used throughout the study or just in this experiment? Why EGFP-PEST rather than EGFP?

Reviewer #2

(Remarks to the Author)
Summary:
In this study, Zhu et al. report the development of multimodal RfxCas13d RNA nucleases whose activity can be precisely controlled by light or small molecules. Through an innovative library screening approach involving random insertions of the photo-switchable AsLOV2 domain, the authors identified a unique insertion site that maintains nuclease activity in the dark and is inactivated upon blue light exposure. Expanding on this, they inserted alternative switchable domains at the same site and demonstrated successful activation in response to light or chemical inducers. The resulting photoswitchable RfxCas13d variants were shown to effectively silence target RNAs in mammalian cells.
This manuscript introduces a highly creative strategy to engineer conditionally active Cas13 tools, offering precise spatiotemporal control over RNA expression. These tools have strong potential for broad application in both basic research and therapeutic contexts. The study is well-executed and conceptually novel, and my comments below are intended to further strengthen the impact and reproducibility of the work.

Major comments:
1) In Figure 2g, light-inactivated RfxCas13d (via the QK634-AsLOV2 insertion) results in partial EGFP silencing within a clonal cell population (middle panel). This suggests that only a subset of cells effectively silence EGFP RNA in the dark. The authors should investigate the underlying cause—potential explanations may include incomplete transfection of the guide RNA or heterogeneous activity of RfxCas13d among cells. Clarifying this issue and optimizing conditions to enhance silencing efficiency across the entire population would strengthen the utility of the system.

2) The use of the UniRapR domain to control Cas13 activity through rapamycin binding is a promising alternative for researchers without optogenetic setups. However, the data in Figure 4c are limited. The authors should expand this section by demonstrating silencing of multiple endogenous transcripts and providing a detailed time-course analysis of RNA knockdown following rapamycin treatment, similar to the kinetics presented for the photoswitchable system in Figure 5.

3) The experiments shown in Figures 4d–4f were conducted in a single clone. As clonal variability can impact phenotypic outcomes, it is standard practice to validate findings in multiple independent clones. The authors are encouraged to replicate these experiments in at least one or two additional clones to reinforce the reproducibility and reliability of the results.

4) In Figure 5, knockdown of endogenous genes is demonstrated at the transcript level via qPCR. To confirm the functional silencing effect, the authors should include complementary protein-level analyses (e.g., Western blotting or flow cytometry) for at least a subset of target genes.

Minor comments:
1) Suppl. Fig 2a: Both NGS graphs are labelled as “before cell incorporation”. I believe the bottom one should be after “cell incorporation”.
2) Most graphs lack statistical significance analyses. Statistical analysis should be performed when relevant
3) For consistency, the author should show non-targeting crRNA under dark and light conditions in Fig 4d-f
4) Figure 5: Authors should add dark vs light for WT-RfxCas13d condition

Version 1:

Reviewer comments:

Reviewer #1

(Remarks to the Author)
My comments have been appropriately addressed and the article is suitable for publication.

Reviewer #2

(Remarks to the Author)
In this revised manuscript, the authors have addressed most of the concerns raised in my previous review, and the overall quality of the manuscript has improved significantly. However, a few minor issues remain and should be addressed.

1) Figure 5 (and Suppl. Fig 12): Although ChemoCas13d shows a clear trend toward silencing endogenous genes upon rapamycin treatment, the effect is variable and often not statistically significant. This limitation is not adequately discussed in the Results or Discussion sections. The authors should explicitly acknowledge the limited and noisy silencing efficacy of ChemoCas13d and its statistical significance in the Results. In contrast, OptoCas13d (Figures 6d-6i) appears to outperform the chemically inducible system in terms of both silencing magnitude and statistical robustness. For clarity, the Discussion should include a brief comparison of the performance and limitations of these two variants.

2) The number of biological replicates should be clearly indicated in all figure legends (e.g., Figures 4d-4g currently do not specify this information).
3) Uncropped Western blot and gel images from N=3 biological replicates should be provided in the Supplementary or Source Data files. The figure legends should include a statement indicating where these uncropped images can be found.

4) Fig 5i: The representative Western blot appears to show enhanced silencing with WT RfxCas13d under light conditions, which is unexpected and does not align with the conclusions. The authors should verify that this blot is correctly labeled. In addition, quantitative analysis of Western blot silencing from N=3 replicates should be included in the main figure rather than in Suppl. Fig 13b.

5) Fig 6c: The graph is missing the OptoCas13d “dark” condition (crGFP), which is shown in Figures 6a and 6b. This condition should be included for consistency.

6) Fig 6a-6c: To show data reproducibility, I recommend adding a bar chart quantifying the silencing efficiency in the FACS plots (% of GFP-positive cells or MFI) based on N=3.

7) Figure 6 caption: (c)-(f) should be replaced by (d)-(i)

Version 2:

Reviewer comments:

Reviewer #2

(Remarks to the Author)
I thank the authors for addressing my comments, and I congratulate them on this innovative paper. The article is suitable for publication.

Open Access This Peer Review File is licensed under a Creative Commons Attribution 4.0 International License, which permits use, sharing, adaptation, distribution and reproduction in any medium or format, as long as you give appropriate credit to the original author(s) and the source, provide a link to the Creative Commons license, and indicate if changes were made.
In cases where reviewers are anonymous, credit should be given to 'Anonymous Referee' and the source.
The images or other third party material in this Peer Review File are included in the article's Creative Commons license, unless indicated otherwise in a credit line to the material. If material is not included in the article's Creative Commons license and your intended use is not permitted by statutory regulation or exceeds the permitted use, you will need to obtain permission directly from the copyright holder.
To view a copy of this license, visit https://creativecommons.org/licenses/by/4.0/

Responses to reviewers shown in blue.

Summary of response:

We thank the reviewers for their thoughtful examination of the paper and helpful suggestions; we really believe that addressing these concerns has improved the data and overall story. Overall, we saw one theme emerge: while both reviewers were convinced that our stimulus-switchable Cas13 variants were functional, they each asked pointed questions about whether the tools reported are *useful*. Can both Chemo- and OptoCas13d be easily used for endogenous gene expression control? How easy is it to generate a cell line exhibiting strong stimulus-dependent knockdown?

To address this question, we have now added an entirely new main text figure and three supplementary figures in which we derive and characterize multiple cell lines expressing the rapamycin-controllable ChemoCas13 system (a new Figure 5 and Supplementary Figures 8-13). In brief, we find that a single clonal sorting step is sufficient to obtain cell lines with strong stimulus-switchable knockdown, an effect that is largely predicted by the level of Cas13 expression in each cell line. We describe this and many other changes in the detailed point-by-point response below.

REVIEWER COMMENTS

Reviewer #1 (Remarks to the Author):

This manuscript describes construction of an optogenetic Cas13 (RfxCas13d), a RNA-targeting CRISPR effector protein, using AsLOV2 insertion. The authors use a screening strategy to identify candidate insertion sites, then test activity of candidates in light and dark for ability to block constitutive EGFP expression to identify a top candidate that shows light-dark differences in function. The authors attempt to characterize mechanistically how the AsLOV2 insertion impacts CRISPR activity, observing minimal effect on complex assembly, implying an effect on nuclease activity, and swap out the AsLOV2 for other regulatory domain insertions.

There are several things done well in this manuscript: it is well-written, incorporates an interesting screen technology, uses rigorous approaches, and the data is convincing. The mechanistic studies add to an understanding of how the insertion site affects activity, and the authors demonstrate the ability to regulate expression at endogenous loci.

While the study seems appropriate for a more specialized journal as is, it has several weaknesses that make it seem premature for publication in a moderately high impact journal such as Nat. Comm. The main limitation is the engineered protein shows only modest light regulation. The authors overcome this limitation by sorting to select cells that optimally express the effector protein at an intermediate level, prior to experimentally testing for light-dark differences, however the requirement for sorting impedes practical use. To improve impact, the authors could incorporate optimization steps (for example, tinkering with the linkers flanking the inserted domains) to try to improve outcomes. As another way to improve impact, applying the system in a model organism could mitigate concerns that the degree of light regulation is insufficient for practical use.

We thank the reviewer for their thoughtful appraisal of the paper. To clarify: we agree that the stimulus-inducible tools only work well over a specific range of protein expression, with constitutive strong knockdown at very high protein levels, and weak knockdown at low protein levels. But within the optimal expression range (spanning about 1 order of magnitude in fluorescence expression on a flow cytometer), we observe potent knockdown, comparable to wild-type constitutive Cas13d. See for example Figure 6c-g (two panels reproduced below) where both a GFP reporter and endogenous gene expression are knocked down comparably to wild-type Cas13d in the light and remain as high as non-targeting cells in the dark:

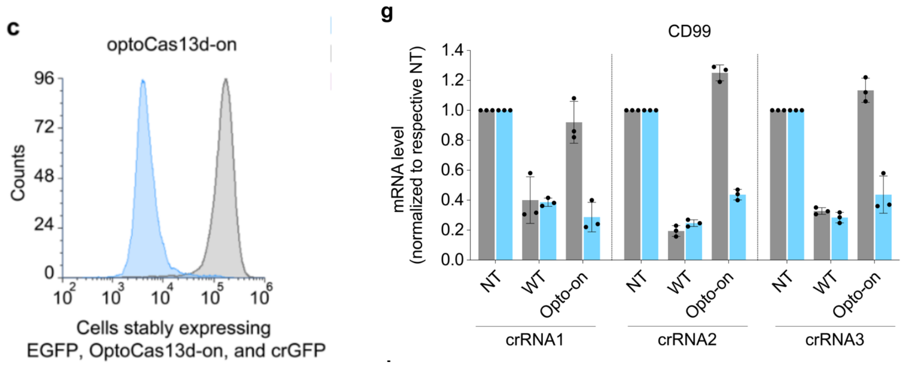

In our experience, cell sorting is a necessary step for obtaining homogenous responses from the vast majority of optogenetic tools (e.g. Cry2 clustering systems, iLID-SSPB dimerization systems) because light can only alter binding affinity over a defined concentration range. Sorting is also commonly employed for CRISPRi and CRISPRa studies. For example, Jonathan Weissman lab's primer on CRISPR studies (https://weissman.wi.mit.edu/crispria_cell_line_primer/) notes that:

"A key parameter for implementing CRISPRi/a systems is construction of cell lines where dCas9 chimera proteins are stably and efficiently expressed in mammalian cells. Although this statement may seem obvious it has been our experience that generating a line where close to 100% of cells in a polyclonal or clonal population stably express dCas9 indefinitely without lentiviral silencing is a crucial aspect of successfully implementing CRISPRi/a systems."

However, we take the reviewer's broader point that we could do a better job showing that our system is *useful* – that it is quite straightforward to obtain a cell line exhibiting high-quality inducible knockdown. It is important to show that our system is robust and that the user does not have to be "lucky" to get it to work.

To address this question, we have explored the generation of ChemoCas13d cell lines in much more detail. We produced 96 clonal cell lines from a broad range of expression levels to characterize the proportion of lines exhibiting different qualities of response. We found that this single cell-sorting step produced robust stimulus-inducible knockdown in 13/96 cell lines comparable to what was obtained from wild-type Cas13d, even without selection for a particular expression window (**Fig. 5C**). Sorting for cell lines within the optimal expression window would make our tool even easier to deploy.

Additional points: Line 227. More explanation of the trans-cleavage assay would be helpful, for example describing how fluorescence is related to activity in this assay. Also, which reference is relevant to this assay? The text refers to ref 29 , while the methods refer to ref 27.

We have now added a more detailed description of the trans-cleavage assay to the Methods (**Lines 736-746**) and carefully checked all references, some of which were corrupted due to incompatibility between reference management software.

Panel 2c, Please indicate the value at the origin of the Y-axis. The manuscript mentions a cell line expressing EGFP but on line 328 notes use of EGFP-PEST. Is EGFP-PEST used throughout the study or just in this experiment? Why EGFP-PEST rather than EGFP?

We apologize for the confusion. Throughout the paper we engineered cells to express a *destabilized* EGFP reporter (EGFP-PEST) to increase the rate at which GFP fluorescence equilibrates in response to a change in stimulus (light or rapamycin addition). We now clarify throughout the text & figures that this GFP variant was used.

Reviewer #2 (Remarks to the Author):

Summary:

In this study, Zhu et al. report the development of multimodal RfxCas13d RNA nucleases whose activity can be precisely controlled by light or small molecules. Through an innovative library screening approach involving random insertions of the photo-switchable AsLOV2 domain, the authors identified a unique insertion site that maintains nuclease activity in the dark and is inactivated upon blue light exposure. Expanding on this, they inserted alternative switchable domains at the same site and demonstrated successful activation in response to light or chemical inducers. The resulting photoswitchable RfxCas13d variants were shown to effectively silence target RNAs in mammalian cells.

This manuscript introduces a highly creative strategy to engineer conditionally active Cas13 tools, offering precise spatiotemporal control over RNA expression. These tools have strong potential for broad application in both basic research and therapeutic contexts. The study is well-executed and conceptually novel, and my comments below are intended to further strengthen the impact and reproducibility of the work.

Major comments:

1) In Figure 2g, light-inactivated RfxCas13d (via the QK634-AsLOV2 insertion) results in partial EGFP silencing within a clonal cell population (middle panel). This suggests that only a subset of cells effectively silence EGFP RNA in the dark. The authors should investigate the underlying cause—potential explanations may include incomplete transfection of the guide RNA or heterogeneous activity of RfxCas13d among cells. Clarifying this issue and optimizing conditions to enhance silencing efficiency across the entire population would strengthen the utility of the system.

This is indeed a crucial point! We realized that we should have presented a crucial piece of data in the original manuscript showing that even wild-type Cas13d generates partial silencing in the assay of **Figure 2G**. We now include this data (reproduced below) as well as a discussion in **Lines 197-211**.

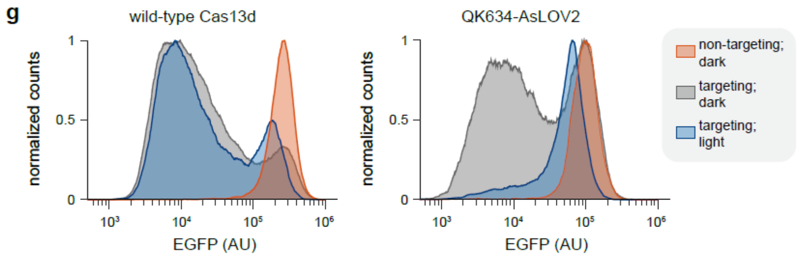

From these data, it is clear that wild-type Cas13 also only exhibits partial EGFP silencing (with some cells still expressing high EGFP levels), likely due to a combination of variability in Cas13 expression levels and heterogeneity in crRNA transfection. (The cell line is only clonal for the EGFP reporter, *not* for crRNA or Cas13 expression.) crRNAs were simply transfected 24 h prior, so some cells likely took up the crRNA and some did not. Cas13 is also only bulk-sorted into the approximate expression range where it exhibits switchable behavior, so it is possible that some variability in EGFP silencing is coming from variation in Cas13 expression levels across cells.

We also show that when all three components are indeed clonally introduced, perfectly unimodal knockdown can indeed be achieved (**Fig. 6C**):

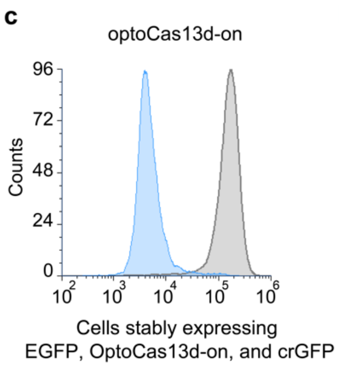

2) The use of the UniRapR domain to control Cas13 activity through rapamycin binding is a promising alternative for researchers without optogenetic setups. However, the data in Figure 4c are limited. The authors should expand this section by demonstrating silencing of multiple endogenous transcripts and providing a detailed time-course analysis of RNA knockdown following rapamycin treatment, similar to the kinetics presented for the photoswitchable system in Figure 5.

We appreciate this excellent suggestion.

We have now added extensive data characterizing the ChemoCas13d system in response to rapamycin treatment. We analyze 96 individual clones that were not pre-selected for specific expression levels, showing that 13/96 clones (14%) exhibit robust knockdown. Endogenous target gene knockdown was robust in all three clones selected for further analysis.

We have refrained from studying the kinetics in this system because it is well established that rapamycin binds very tightly to its target protein domains, leading to slow dissociation kinetics even after multiple washes. While the ChemoCas13d system is likely to be useful for acute

inducible control over RNA knockdown, we would argue that it is not ideal for rapidly reversible control. We now mention this point in the Discussion (**Lines 493-495**).

3) The experiments shown in Figures 4d–4f were conducted in a single clone. As clonal variability can impact phenotypic outcomes, it is standard practice to validate findings in multiple independent clones. The authors are encouraged to replicate these experiments in at least one or two additional clones to reinforce the reproducibility and reliability of the results.

We agree that this is important. We decided to address the question of clonal variability comprehensively in the case of our new ChemoCas13 data. We now characterize the performance of 96 clones and follow up extensively on three independent clones, showing similar performance in each (and similar performance to what we previously showed from the OptoCas13d-on system). These new results have been added to **Figure 5** of the manuscript.

4) In Figure 5, knockdown of endogenous genes is demonstrated at the transcript level via qPCR. To confirm the functional silencing effect, the authors should include complementary protein-level analyses (e.g., Western blotting or flow cytometry) for at least a subset of target genes.

We have now demonstrated protein-level knockdown for one target protein, CLTA, using the ChemoCas13d system (**Fig 5l**, **Supplementary Fig. 13**, and **Lines 409-416**).

Minor comments:

1) Suppl. Fig 2a: Both NGS graphs are labelled as “before cell incorporation”. I believe the bottom one should be after “cell incorporation”.

Thank you for your sharp eyes! This has now been corrected.

2) Most graphs lack statistical significance analyses. Statistical analysis should be performed when relevant

We now present t-test comparisons between light and dark condition for all endogenous gene knockdown experiments.

3) For consistency, the author should show non-targeting crRNA under dark and light conditions in Fig 4d-f

Non-targeting guide comparisons have now been added throughout.

4) Figure 5: Authors should add dark vs light for WT-RfxCas13d condition

Done.

Responses to reviewers shown in blue.

REVIEWER COMMENTS

Reviewer #1 (Remarks to the Author):

My comments have been appropriately addressed and the article is suitable for publication.

We thank the reviewer for their support!

Reviewer #2 (Remarks to the Author):

In this revised manuscript, the authors have addressed most of the concerns raised in my previous review, and the overall quality of the manuscript has improved significantly. However, a few minor issues remain and should be addressed.

1) Figure 5 (and Suppl. Fig 12): Although ChemoCas13d shows a clear trend toward silencing endogenous genes upon rapamycin treatment, the effect is variable and often not statistically significant. This limitation is not adequately discussed in the Results or Discussion sections. The authors should explicitly acknowledge the limited and noisy silencing efficacy of ChemoCas13d and its statistical significance in the Results. In contrast, OptoCas13d (Figures 6d-6i) appears to outperform the chemically inducible system in terms of both silencing magnitude and statistical robustness. For clarity, the Discussion should include a brief comparison of the performance and limitations of these two variants.

This is a fair point and we now mention this point in the Discussion (Lines 406-409; 477-480).

2) The number of biological replicates should be clearly indicated in all figure legends (e.g., Figures 4d-4g currently do not specify this information).

We appreciate the reviewer for the suggestion. We have added the number of biological replicates (n = 3 biological replicates) in the figure caption for Figure 4d-g and checked other figure legends to make sure the number of replicates are specified.

3) Uncropped Western blot and gel images from N=3 biological replicates should be provided in the Supplementary or Source Data files. The figure legends should include a statement indicating where these uncropped images can be found.

We thank the reviewer for the suggestion. We have now added uncropped Western blots for all three biological replicates in Supplementary Figure S13. Crucially, taking a second look at the Westerns led us to identify bubbles in transfer for the loading control gel in one of our replicates; we re-ran those gels and re-quantified the data, leading to moderately cleaner results.

4) Fig 5i: The representative Western blot appears to show enhanced silencing with WT RfxCas13d under light conditions, which is unexpected and does not align with the conclusions. The authors should verify that this blot is correctly labeled. In addition,

quantitative analysis of Western blot silencing from N=3 replicates should be included in the main figure rather than in Suppl. Fig 13b.

We thank the reviewer for these careful observations. We have re-verified the original raw data and confirmed all samples in Fig. 5i are correctly labeled. We agree that the modest level of knockdown and variability between replicates does make it difficult to draw very strong conclusions for every comparison, something we now point out in the main text (as also suggested by the reviewer in Comment #1 above). Importantly, re-quantification (after re-running the lysates for one replicate with a bubble in the actin control) has resulted in a moderately cleaner result, with more comparable CTLA levels the -rap and +rap wild-type Cas13 cases.

In response to the reviewer's suggestion, we have now moved the quantitative densitometry analysis from Supplementary Fig. S13 to Fig. 5i. The updated panel shows mean ± s.d. from three independent experiments with statistical tests.

5) Fig 6c: The graph is missing the OptoCas13d "dark" condition (crGFP), which is shown in Figures 6a and 6b. This condition should be included for consistency.

We thank the reviewer for this suggestion. In Fig. 6c, we used a clonal line with stable integration of the EGFP-targeting crRNA (crGFP) together with OptoCas13d-on to eliminate heterogeneity arising from variable crRNA delivery. Because the targeting crRNA is stably integrated and constitutively expressed in this cell line, a non-targeting (NT) crRNA control is not technically possible. Fig. 6c is intended to demonstrate light-dependent robust EGFP knockdown response upon stable co-expression of OptoCas13d-on and a corresponding crGFP.

6) Fig 6a-6c: To show data reproducibility, I recommend adding a bar chart quantifying the silencing efficiency in the FACS plots (% of GFP-positive cells or MFI) based on N=3.

We appreciate the reviewer for this helpful suggestion. To quantify reproducibility and silencing efficiency, we have added a new panel (Fig. 6d) showing the fraction of $EGFP^+$ cells across a minimum of three independent biological replicates (n >= 3) under the indicated conditions. This bar graph provides quantitative assessment of knockdown efficiency corresponding to the representative FACS plots shown in Fig. 6a–c. The figure legend and Results section have been updated accordingly.

7) Figure 6 caption: (c)-(f) should be replaced by (d)-(i).

We thank the reviewer for catching the mistake. We have fixed the Figure 6 caption.
